# Epigenomic and metabolic responses of hypothalamic POMC neurons to gestational nicotine exposure in adult offspring

**DOI:** 10.1186/s13073-016-0348-2

**Published:** 2016-09-08

**Authors:** Jose P. Silva, Guerline Lambert, Derek van Booven, Claes Wahlestedt

**Affiliations:** 1 Department of Psychiatry and Behavioral Sciences and Center for Therapeutic Innovation, Miller School of Medicine, University of Miami, Miami, FL 33136 USA; 2 John P. Hussman Institute for Human Genomics, Miller School of Medicine, University of Miami, Miami, FL 33136 USA

**Keywords:** POMC neurons, Leptin-melanocortin signaling, Gestational nicotine exposure, Obesity, Type 2 diabetes, Epigenomics, Transcriptomics, lncRNA, Gm15851

## Abstract

**Background:**

Epidemiological and animal studies have reported that prenatal nicotine exposure (PNE) leads to obesity and type-2 diabetes in offspring. Central leptin-melanocortin signaling via hypothalamic arcuate proopiomelanocortin (POMC) neurons is crucial for the regulation of energy and glucose balance. Furthermore, hypothalamic POMC neurons were recently found to mediate the anorectic effects of nicotine through activation of acetylcholine receptors. Here, we hypothesized that PNE impairs leptin-melanocortinergic regulation of energy balance in first-generation offspring by altering expression of long non-coding RNAs (lncRNAs) putatively regulating development and/or function of hypothalamic POMC neurons.

**Methods:**

C57BL/6J females were exposed ad libitum to nicotine through drinking water and crossed with C57BL/6J males. Nicotine exposure was sustained during pregnancy and discontinued at parturition. Offspring development was monitored from birth into adulthood. From the age of 8 weeks, central leptin-melanocortin signaling, diabetes, and obesity susceptibility were assessed in male offspring fed a low-fat or high-fat diet for 16 weeks. Nicotine-exposed and non-exposed C57BL/6J females were also crossed with C57BL/6J males expressing the enhanced green fluorescent protein specifically in POMC neurons. Transgenic male offspring were subjected to laser microdissections and RNA sequencing (RNA-seq) analysis of POMC neurons for determination of nicotine-induced gene expression changes and regulatory lncRNA/protein-coding gene interactions.

**Results:**

Contrary to expectation based on previous studies, PNE did not impair but rather enhanced leptin-melanocortinergic regulation of energy and glucose balance via POMC neurons in offspring. RNA-seq of laser microdissected POMC neurons revealed only one consistent change, upregulation of *Gm15851*, a lncRNA of yet unidentified function, in nicotine-exposed offspring. RNA-seq further suggested 82 *cis*-regulatory lncRNA/protein-coding gene interactions, 19 of which involved coding genes regulating neural development and/or function, and revealed expression of several previously unidentified metabolic, neuroendocrine, and neurodevelopment pathways in POMC neurons.

**Conclusions:**

PNE does not result in obesity and type 2 diabetes but instead enhances leptin-melanocortinergic feeding and body weight regulation via POMC neurons in adult offspring. PNE leads to selective upregulation of *Gm15851*, a lncRNA, in adult offspring POMC neurons. POMC neurons express several lncRNAs and pathways possibly regulating POMC neuronal development and/or function.

**Electronic supplementary material:**

The online version of this article (doi:10.1186/s13073-016-0348-2) contains supplementary material, which is available to authorized users.

## Background

The adipocyte-secreted hormone leptin has critical anorectic and body weight-lowering actions that are mediated by the central melanocortin system [1, 2]. The melanocortin system consists of first order signaling neurons in the hypothalamic arcuate nucleus comprising neuropeptide Y (NPY) and Agouti-related protein (AgRP) co-expressing neurons and of proopiomelanocortin (POMC)-expressing neurons. These first order signaling neurons project to second order signaling neurons expressing melanocortin 3 and 4 receptors (MC3/4-Rs) located in several hypothalamic and extrahypothalamic areas, where they exert anorectic actions, including the periventricular nucleus (PVN), a satiety center, and the lateral hypothalamic area (LHA), a feeding center. POMC is a precursor polypeptide for α-melanocyte-stimulating hormone (α-MSH). α-MSH acts as an agonist to MC3/4-Rs of second order signaling neurons in the PVN to inhibit food intake and increase energy expenditure. AgRP is an antagonist of α-MSH at MC3/4-Rs and potent activator of food intake and body weight gain. Leptin regulates transcription of the genes encoding POMC and AgRP. Leptin binds to leptin receptors expressed in POMC and NPY/AgRP neurons. This triggers phosphorylation and activation of Janus kinase 2 (JAK2), which in turn phosphorylates signal transducer and activator of transcription 3 (P-STAT3). P-STAT3 homodimerizes and translocates to the nucleus to promote transcription of the POMC gene and inhibit transcription of the AgRP gene [3]. Leptin also stimulates release of α-MSH from POMC neurons [4] and inhibits release of AgRP from NPY/AgRP neurons [5]. Independently of decreasing food consumption and body weight, leptin signaling via hypothalamic POMC neurons potently decreases glycemia and increases locomotor activity, thereby preventing hyperglycemia and normalizing physical activity of morbidly obese, severely diabetic, and hypoactive, leptin-receptor deficient *Lepr*^db/db^ mice [6].

Mutations of the leptin, leptin receptor, POMC, and MC4-R genes lead to severe obesity in rodents and in humans, underscoring the importance of central leptin-melanocortin signaling for the regulation of energy balance [7–9]. Furthermore, common human obesity is frequently associated with leptin resistance characterized by an inability of leptin to decrease body weight.

Nicotine has anorectic effects. One underlying mechanism is the activation of hypothalamic POMC neurons by nicotinic α3β4 acetylcholine receptors and the subsequent activation of MC4-R expressing target neurons in the PVN [10]. The metabolic consequences of gestational nicotine exposure in first-generation offspring have been investigated in human epidemiological studies [11–14] and rodent models [15–18]. These studies concluded that gestational nicotine exposure increases the risk for obesity and type 2 diabetes in offspring. Furthermore, it was reported that in utero or early postnatal nicotine exposure upregulates POMC mRNA in neonate rhesus macaque [19, 20]. These observations led us to hypothesize that gestational nicotine exposure impacts leptin-melanocortinergic regulation of energy balance in first-generation offspring.

More than 70 % of the mammalian genome is transcribed as non-coding RNAs (ncRNAs) of various sizes ranging from 20 nucleotides to over 100 kb [21, 22]. NcRNAs are subdivided into short and long ncRNAs (lncRNAs), which are shorter and longer than 200 nucleotides, respectively [23], as well as processed transcripts, which do not contain an open reading frame, do not contain retained introns, and cannot be placed in the short and long ncRNA group. Similar to protein-coding mRNAs, lncRNAs can be spliced, polyadenylated, and capped [23]. LncRNAs are subdivided into: (1) antisense RNAs, which are transcribed from the opposite DNA strand of a protein-coding gene overlapping its intronic and exonic sequences; (2) long intergenic non-coding RNAs (lincRNA) transcribed from intergenic DNA regions; (3) sense-intronic RNAs transcribed from introns of a protein-coding gene on the same DNA strand with no overlap of exonic sequence; (4) sense-overlapping RNAs containing a protein-coding gene in one of its introns on the same DNA strand with no overlap of exonic sequence; and (5) 3′-overlapping ncRNAs, which are transcribed from the 3′-untranslated region of a larger gene. LncRNAs are predominantly localized in the nucleus at usually much lower expression levels than mRNAs.

While lncRNAs were initially believed to represent transcriptional noise, studies in recent years have revealed developmental-, tissue- and cell-type-specific expression of lncRNAs [24, 25] and regulatory roles in important biological processes such as X-chromosome inactivation in females, silencing of tumor suppressor genes, mediation of DNA damage and cellular stress responses, gene imprinting, heterochromatin spreading across DNA insulator sequences, regulation of stem cell pluripotency, cell fate specification, and neural development [26–30]. However, although many different biological functions have been assigned to lncRNAs, most lncRNAs lack functional annotations. LncRNA genes show poorer sequence conservation than protein-coding genes although well-conserved lncRNAs with biologically important functions exist [31]. The number of lncRNA genes has dramatically increased in late evolution and appears to scale with genome size. Meanwhile, the number of protein-coding genes has not markedly increased during late evolution. Therefore, one emergent hypothesis is that the increase in lncRNA genes contributes to regulatory and organismal complexity [32]. LncRNAs can regulate expression of flanking or overlapping coding genes (“*cis*-regulation”) or far distant coding genes located on the same or another chromosome (“*trans*-regulation”) in a positive (concordant) or negative (discordant) manner [25, 33, 34]. LncRNA-guided gene regulation involves epigenetic, transcriptional, and post-transcriptional mechanisms. LncRNAs are thought to provide scaffolds for histone methyltransferases, histone acetylases, and DNA methyltransferases [26, 27], and can regulate splicing, editing, or degradation of protein-coding RNAs [26].

Here we interrogated whether PNE alters expression of lncRNAs with putative roles in hypothalamic POMC neuronal development/function and leptin-melanocortinergic regulation of energy balance in first-generation offspring in a mouse model of human maternal nicotine exposure from adolescence until parturition. We found that PNE does not impair but rather enhances leptin-melanocortinergic regulation of energy balance in first-generation offspring and selectively upregulates expression of *Gm15851*, a lncRNA of yet unidentified function, in hypothalamic POMC neurons. Furthermore, we report expression of several signaling pathways and lncRNAs that might regulate development and function of hypothalamic POMC neurons.

## Methods

### Animals

Animals used in the study were from the C57BL/6J mouse strain (RRID:IMSR_JAX:000664, Jackson Laboratories, Bar Harbor, ME, USA) or transgenic mice expressing the enhanced green fluorescent protein (EGFP) in hypothalamic POMC neurons (C57BL/6J-Tg(Pomc-EGFP)1Low/J, RRID:IMSR_JAX:009593, Jackson Laboratories, Bar Harbor, ME, USA). Metabolic, hormonal, hypothalamic gene expression and leptin-signaling studies were conducted in C57BL/6J male offspring. RNA sequencing (RNA-seq) studies of laser microdissected POMC neurons were conducted in POMC-EGFP transgenic male offspring. POMC-EGFP transgenic offspring were tail biopsied at postnatal day 17 and genotyped using established PCR protocols provided by the Jackson Laboratories. Offspring were weaned at postnatal day 21 and kept in groups of 3–4 mice per cage. Male offspring were singly housed with environmental enrichment from the age of 8 weeks onwards until the end of the study. All mice were housed in a pathogen-free barrier animal facility and kept in a temperature-controlled (22 ± 0.5 °C) and humidity-controlled (50 %) animal room on a 12 h light/dark schedule with light on at 07:00 and free access to food and water.

### Prenatal nicotine exposure

Six-week-old females were treated for 4 weeks ad libitum with drinking water containing 200 μg/mL nicotine hydrogen tartrate sweetened with 2 % saccharin (w/v), pH 7.4 [35]. Controls were administered pH-matched drinking water containing the equivalent amount of tartaric acid and 2 % saccharin (w/v). The inclusion of saccharin was required because nicotine in the drinking water causes taste-aversion [36, 37] and reduces maternal fluid intake [35]. Drinking solutions were changed twice a week. At the age of 10 weeks, females were mated to unexposed males. Nicotine administration continued throughout mating and pregnancy and was discontinued at parturition. Mothers were allowed only one pregnancy.

### Determination of plasma cotinine

Plasma cotinine levels were determined using a Cotinine (Mouse/Rat) enzyme linked immunosorbent assay kit (Abnova, Walnut, CA, USA) following the manufacturer’s directions. All samples were assayed in duplicates. The intra-assay coefficient of variation (CV) of the cotinine ELISA measurements was 3.9 %.

### Diets

All animals were fed a standard (low-fat) diet (STD) with a metabolizable energy density of 3.1 kcal/g derived from 28.7 % kcal protein, 58.53 % kcal carbohydrates, and 12.73 % kcal fat (5010, LabDiet, St Louis, MO, USA). A subset of male offspring was subjected to a high-fat diet (HFD) with a metabolizable energy density of 4.7 kcal/g derived from 17 % kcal protein, 43 % kcal carbohydrates, and 41 % kcal fat, containing 0.21 % (w/w) Cholesterol (Western Diet, Research Diets, Inc., New Brunswick, NJ, USA) starting from the age of 8 weeks.

### Determination of energy balance

Body composition (body fat content, lean mass, and body fluid) was determined by nuclear magnetic resonance (minispec TD-NMR Analyzer, Bruker Optics, Inc., Billerica, MA, USA). Food consumption of singly housed animals was measured twice a week. Fresh food pellets were provided twice a week to avoid temperature-dependent spoilage. Any residual bits of food in the bedding were included in the measurements. A comprehensive laboratory animal monitoring system (Columbus Instruments, Columbus, OH, USA) determined oxygen consumption, carbon dioxide production, heat production, locomotor activity, and food intake simultaneously in individual animals. Measurements were taken in metabolic cages placed in a temperature-controlled enclosure set at 23.5 °C. Mice were acclimated to the metabolic cages for 72 h prior to measurements for an additional 72 h.

### Determination of glucose balance

Blood glucose measurements were carried out with a OneTouch glucometer (LifeScan, Inc.). Blood glucose and insulin concentrations were determined by tail venipuncture and submandibular bleeding, respectively, between 09:00 and 10:00 following 14 h of fasting. Insulin was measured by enzyme linked immunosorbent assay (Ultra Sensitive Mouse Insulin ELISA Kit, Crystal Chem, Inc., Chicago, IL, USA). All samples were assayed in duplicates. The intra-assay CV of the insulin ELISA measurements was 9.9 %. Glucose tolerance tests (GTTs) were conducted in 14 h fasted mice by injecting i.p. glucose 1 g/kg body weight. Insulin tolerance tests (ITTs) were conducted in 6 h fasted mice by injecting i.p. Insulin (Humulin R, Eli Lilly, Indianapolis, IN, USA) 0.5 IU/kg body weight. Blood glucose concentrations for the GTT and ITT were measured from the tail tip. Calculating the area under the curve (AUC) of glucose concentration-time point graphs using Prism 5 (RRID:SCR_002798, GraphPad Software, La Jolla, CA, USA) quantitatively assessed glucose and insulin tolerance.

### Determination of plasma lipids

All plasma lipids were determined using VITROS**®** Chemistry Products and analyzer (Ortho-Clinical Diagnostics, Inc., Rochester, NY, USA). Total cholesterol (Tot Chol) was measured by the VITROS CHOL Slide method. High-density lipoprotein cholesterol (HDL-C) was determined by the VITROS direct HDL-C (dHDL) slide assay. Triglycerides were measured by the VITROS TRIG Slide method. Low density lipoprotein cholesterol (LDL-C) and Very low density lipoprotein cholesterol (VLDL-C) concentrations were calculated as follows: VLDL-C = TG/5; LDL-C = Tot Chol – HDL-C – VLDL-C.

### Refeeding experiments and determination of Leptin and MTII sensitivity

Refeeding experiments were conducted as described previously [38]. Mice were fasted overnight for 12 h and then injected i.p. leptin 5 mg/kg (A.F. Parlow National Hormone and Peptide Program, Torrance, CA, USA), Melanotan II (MTII) 5 mg/kg (Tocris, Ellisville, MO, USA), or the equivalent volume of vehicle (phosphate buffered saline (PBS)). Body weight was determined prior to injection, as well as 1 h, 2 h, 4 h, 8 h, and 24 h post injection. A known amount of food was added to each cage immediately after injection and food intake was measured 1 h, 2 h, 4 h, 8 h, and 24 h post injection. Mice had unlimited access to water during the entire experiment. Plasma leptin concentrations were determined by ELISA (Mouse Leptin ELISA Kit, Crystal Chem, Inc., Chicago, IL, USA). All samples were assayed in duplicates. The intra-assay CV for the leptin ELISA measurements was 5.1 %.

### Immunofluorescence detection and quantification of leptin-induced nuclear STAT3 phosphorylation

Mice were fasted overnight for 14 h and injected i.p. leptin 5 mg/kg (*n* = 3) or the equivalent volume of vehicle (PBS) (*n* = 2–3). Thirty minutes post injection, animals were perfused with 10 % formalin under general anesthesia. Brains were post-fixed in 10 % formalin for 24 h at 4 °C and paraffine-embedded. Coronal sections were cut at 6 μm, deparaffinized, and rehydrated through sequential washes in Xylene, 95 % ethanol, 70 % ethanol, and water. Sections were permeabilized with PBS/0.3 % Triton X-100 for 10 min and blocked with 5 % normal goat serum in PBS/0.3 % Triton X-100 for 1 h at room temperature. Sections were incubated overnight at 4 °C with mouse monoclonal Phospho-STAT3 (Tyr 705) antibody (Cell Signaling Technology, Danvers, MA, USA; Cat# 4113S, RRID: AB_2198588) diluted 1:50 and rabbit anti-POMC antibody (Phoenix Pharmaceuticals, Inc., Burlingame, CA, USA; Cat# H-029-30, RRID: AB_2307442) diluted 1:300 in 2 % goat serum/PBS/0.3 % Triton X-100. Another set of sections was incubated with rabbit monoclonal Phospho-STAT3 (Tyr 705) (D3A7) antibody (Cell Signaling Technology, Danvers, MA, USA; Cat# 9145S, RRID:AB_561305) diluted 1:100 in 1 % BSA/PBS/0.3 % Triton X-100. Sections were washed with PBS and then incubated with anti-rabbit IgG and anti-mouse IgG antibodies conjugated with the Alexa fluorochromes 488 and 555, respectively. After rinsing sections with PBS, Prolong® Gold Antifade Reagent (LifeTechnologies, Grand Island, NY, USA) was added and coverslips were mounted. Sections were analyzed on an inverted Leica Microsystems SP5 imaging system with an XYZ automated stage and AOBS wavelength controls for laser tuning. The system was equipped with a set of lasers providing the following laser lines: 405, 458, 476,488, 496, 514, 561, and 633 nm. The system ran under the Leica Application Suite (LAS) AF 2.7.1 software. Images were acquired using a 20× dry lens with a numerical aperture of 0.7. Images were acquired from 3–4 matched (rostral-caudal) hypothalamic sections encompassing the bregma coordinates −1.34 mm and −2.06 mm [39]. P-STAT3 reactive POMC neurons were identified by co-localization of nuclear P-STAT3 and cytosolic POMC-polypeptide [38]. Differences in leptin-induced P-STAT3 signaling were assessed by comparing average counts of P-STAT3-positive ARC neurons or P-STAT3-positive POMC neurons in brain sections of individual mice (*n* = 3 mice per group).

### Determination of hypothalamic POMC mRNA expression

Total RNA was isolated from microdissected whole hypothalami using the RNeasy Mini kit (Qiagen). Total RNA (500 ng) was reverse transcribed (Superscript III first strand cDNA synthesis kit; Invitrogen, ThermoFisher Scientific). Real-time PCR reactions were carried out on an ABI 7900HT light cycler using the Taqman Universal Master Mix and validated gene-specific Taqman expression assays for proopiomelanocortin and β-actin (Applied Biosystems, ThermoFisher Scientific). POMC mRNA was normalized to β-actin mRNA by the ΔΔCt method.

### Laser micro-dissections and RNA extractions of hypothalamic POMC neurons

Prenatally nicotine-exposed and non-exposed transgenic adult male mice expressing the enhanced green fluorescent protein in hypothalamic POMC neurons [40] were euthanized in week 10 of the STD by CO_2_ inhalation followed by cervical dislocation and decapitation. Brains were quickly removed, fresh-frozen in dry ice, and stored at −80 °C. Brains were sectioned on a cryostat Leica CM 3050 S at 15 μm. Sections were attached onto Director® slides (Expression Pathology, Inc., Rockville, MD, USA), dehydrated in acetone for 1 min at room temperature, and desiccated for 2 min in a closed petri-dish containing dessicant (Drierite). Slides were immediately placed upside-down on the stage of a laser microdissection microscope Leica AS LMD equipped with a HCX PL Fluotar 20×/0.4 NA lens and a Hitachi HV-C20A camera. POMC-GFP neurons in the mediobasal hypothalamus were visualized by the green fluorescent appearance of their cell bodies, which were collected directly in 20 μL lysis buffer of the RNAequous microextraction kit (Life Technologies, ThermoFisher Scientific). Collections from individual sections were not allowed to continue for more than 30 min. Lysates were immediately frozen on dry ice and stored at −80 °C. Between 400 and 600 POMC neurons were isolated from the entire hypothalamus of each mouse brain. RNA was isolated following the directions provided in the RNAequous microextraction kit. RNA samples were run on an electropherogram (2100 Bioanalyzer, Agilent Technologies) for determination of RNA quality, concentration, and yield. Samples with RNA integrity numbers (RIN) above 6 were used for generation of RNA-seq libraries.

### RNA sequencing

A range of 1–2 offspring coming from five different nicotine-exposed dams (*n* = 6 PNE offspring total) and 1–2 offspring coming from four different non-exposed dams (*n* = 5 control offspring total) were subjected to RNA-seq. Directional RNA-seq libraries were generated from﻿ a range of 200–400 pg total RNA using the Ovation Single Cell RNA-seq kit (NuGen, San Carlos, CA, USA) following the manufacturer’s directions. All RNA-seq libraries were run on an electropherogram (2100 Bioanalyzer, Agilent Technologies) to confirm the expected fragment size distribution. Libraries were paired-end sequenced on Illumina’s HiSeq2000 sequencer. Of all read-pairs, 90–95 % passed Illumina’s quality filters. For subsequent alignments, the first eight nucleotides of the forward read were trimmed according to the manufacturer’s directions for analysis of Ovation Single Cell RNA-seq libraries. Pass-filter read-pairs were aligned to the mouse reference mm10 genome using STAR [41]. Read-pairs aligning to the genome with more than two mismatches or aligning to more than one site of the genome were discarded from further analyses. The aligned read-pairs were run through HTSeq (RRID:SCR_005514) for transcript quantification against the GENCODE reference gtf file vM4 for the mouse mm10 genome [42]. After all features had been quantified the data was run through three differential expression calculators in edgeR (RRID:SCR_012802) [43], DESeq (RRID:SCR_000154) [44], and baySeq (RRID:SCR_012795) [45]. The intersection of the three methods was taken and transformed into a list of the final differentially expressed features. Differentially expressed features were determined by cutoff adjusted *p* values of 0.05 across all three methods. For heatmap generation, the default parameters of the heatmap.2 function within the R package gplots v2.16.0 (https://cran.r-project.org/web/packages/gplots/index.html) were used. Function and expression annotations for coding and non-coding genes were retrieved from the Protein ANalysis THrough Evolutionary Relationships (PANTHER) Classification System (RRID:SCR_004869) [46]. Expression differences of Gm15851 were confirmed by strand-specific qRT-PCR in the same RNA samples used for RNA-seq (*n* = 6 PNE offspring; *n* = 5 control offspring). Following quantitation of RNA concentrations by the RNA 6000 pico assay on an Agilent 2100 Bioanalyzer (Agilent Technologies), 500 pg of total RNA was reverse transcribed using a strand-specific primer (sequence: TCCTGGATCTGCAGCACAATCG) annealing to the *Gm15851* transcript and the superscript IV first strand synthesis system (ThermoFisher Scientific). Reaction conditions followed the manufacturer’s directions. After cDNA synthesis, the RNA template was digested with RNAse H. Real-time PCR reactions were run in triplicate using a custom-built Taqman gene expression assay for *Gm15851* (forward primer sequence: CCGGCACGTTGCTGATC; reverse primer sequence: CTCCTTCAACATCTCCAACTTGCT; Taqman reporter sequence: CCACCTGTCTCACAACAA) (ThermoFisher Scientific) on a QuantStudio 7 Flex real time PCR system (ThermoFisher Scientific). Ct values were determined with QuantStudio software (ThermoFisher Scientific). Dilutions of an expression plasmid containing the cDNA sequence of *Gm15851* were assayed in parallel to generate a reference standard curve and quantitate the relative amounts of *Gm15851* in each sample.

### Data analysis

Study groups consisted of *n* = 7–9 male offspring unless otherwise indicated. Male offspring were selected from *n* = 6 nicotine-exposed and *n* = 7 non-exposed litters. Male offspring in each study group originated from *n* = 3–4 different dams. Statistical analyses were conducted with GraphPad Prism 5 (RRID:SCR_002798, GraphPad Software, La Jolla, CA, USA). Data were subjected to various normality tests (Kolmogorov–Smirnov, D’Agostino–Pearson Omnibus, and Shapiro–Wilk) prior to performing the following parametric or non-parametric statistical tests: two-tailed unpaired *t* test, Welch’s test for uneven variances, and Mann–Whitney test for two-group comparisons; Fisher’s exact test for contingency analyses of postnatal survival; two-way ANOVA for determination of the effect of PNE and diet on metabolic, hormonal, and gene expression parameters, and the combined effect of PNE and leptin/MTII on body weight gain and food intake in refeeding experiments; two-way repeated measures (RM) ANOVA for determination of the effect of nicotine on maternal fluid intake, food intake, and body weight before and during gestation and the effect of PNE on glucose and insulin tolerance; Bonferroni post-tests following ANOVA for two-group comparisons. The α-value was set at 0.05 for each statistical test. Data were expressed as mean ± SEM.

## Results

### Prenatal nicotine exposure and early postnatal development of offspring used in the study

**Fig. 1:**
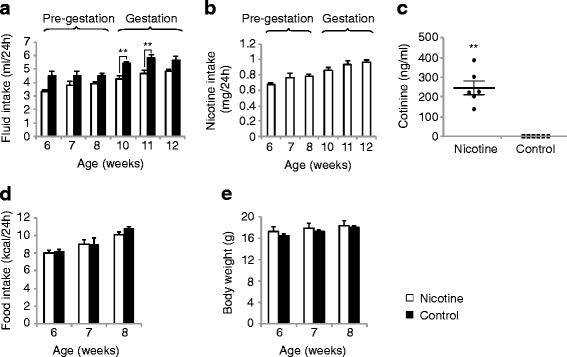
Ad libitum nicotine intake led to high plasma cotinine levels, moderately reduced fluid intake, and did not alter body weight or food consumption of dams. **a** Twenty-four-hour fluid intake was determined in group-housed females (*n* = 4 cages of 3 females/group) before pregnancy and in individually housed females (*n* = 10/group) during gestation. **b** Nicotine intake before (*n* = 4) and during pregnancy (*n* = 10) was calculated based on 24-h fluid intake. **c** Plasma cotinine levels were determined by enzyme-linked immunosorbent assay (ELISA) after 4 weeks of nicotine ingestion. Plasma cotinine was detected in all nicotine-exposed females (*n* = 6) and was undetectable in non-exposed females (*n* = 6). **d** Twenty-four-hour food intake was determined in group-housed females (*n* = 4 cages of 3 females/group) before pregnancy. **e** Body weight was determined weekly before pregnancy (*n* = 12/group). Data were analyzed by two-way RM ANOVA followed by Bonferroni post-test (**a**) or by Mann–Whitney test (**c**) (**, *p* < 0.01). All data are expressed as mean ± SEM

C57BL/6J females were exposed to nicotine ad libitum through drinking water containing nicotine hydrogen tartrate salt at a concentration of 200 μg/mL. Controls received drinking water containing the equivalent amount of pH-matched tartaric acid. Nicotine exposure started at the age of 6 weeks, continued throughout mating at the age of 10 weeks and gestation, and ended at parturition. Since nicotine causes taste aversion [36, 37], the drinking water of both, nicotine-exposed and non-exposed dams was sweetened with 2 % (w/v) saccharin [35]. Nicotine moderately reduced weekly fluid volume intake before and during gestation (Pre-Gestation: F_1,12_ = 5.41, *p* = 0.06; Gestation: F_1,36_ = 23.4, *p* = 0.0001; RM ANOVA) (Fig. 1a). The mean daily water intake of nicotine-exposed females (≈2 mL/10 g body weight/24 h at 6–8 weeks of age) remained moderately above the mean values reported for wild-type mice of unspecified sex, strain, and age (1.5 mL/10 g body weight/24 h) and those reported for C57BL/6 females aged 7–9 weeks (1.64 mL/10 g body weight/24 h, Mouse Phenome Data Base, The Jackson Laboratory). Mean daily nicotine ingestion calculated based on fluid intake was 0.77 ± 0.03 mg/day (*n* = 4) before pregnancy and 0.92 ± 0.03 mg/day (*n* = 10) during pregnancy (Fig. 1b). Moreover, the plasma levels of cotinine, a metabolite of nicotine, and indicator of tobacco smoke exposure in humans [47] determined after 4 weeks of ad libitum nicotine intake were in the range of 137.4–385.1 ng/mL (mean value: 245 ± 0.03 ng/mL; *n* = 6) (Fig. 1c). These plasma cotinine values are within the range reported in humans who smoke 15–24 cigarettes/day and > 25 cigarettes/day and exhibit serum cotinine concentrations of 230–280 ng/mL and 260–300 ng/mL, respectively [48]. Plasma cotinine was undetectable in age-matched non-exposed dams. Ad libitum nicotine ingestion had no significant impact on maternal food intake (F_1,12_ = 3.04, *p* = 0.13; RM ANOVA) (Fig. 1d) or maternal body weight (F_1,44_ = 3.4, *p* = 0.08; RM ANOVA) (Fig. 1e) as reported for the C57BL/6 mouse strain [35]. Dams were allowed only one pregnancy. Nicotine-exposed and non-exposed litters displayed no significant difference in mean litter size at postnatal day (PD)1 (PNE: 6.7 ± 0.37; control: 6.1 ± 0.3; *n* = 10 litters/group; t_18_ = 1.24, *p* = 0.23; *t* test). Postnatal survival of PNE offspring tended to be more compromised than that of control offspring (62.7 % versus 73.8 %) by PD21 but contingency analysis of alive and dead PD21 offspring revealed no significant differences between groups (*p* = 0.19 by two-sided Fisher’s exact test) and litter sizes were the same in both groups by PD21 (PNE: 4.2 ± 1.02; control: 4.5 ± 0.8; *n* = 10 litters/group; t_18_ = 0.22, *p* = 0.82; *t* test). However, PNE led to moderate decreases in mean body weight (PNE: 6.55 ± 0.23 g; control: 7.16 ± 0.14 g; *n* = 42–45/group; *p* = 0.027; Mann–Whitney test) and mean crown-rump length (PNE: 5.77 ± 0.12 cm; control: 6.15 ± 0.45 cm; *n* = 42–45/group; *p* = 0.037; Mann–Whitney test) of PD21 offspring. Therefore, to exclude differences in maternal care as a confounding factor in the assessment of the long-term metabolic consequences of PNE, we selected for the metabolic studies six nicotine-exposed litters and seven non-exposed litters showing similar postnatal survival rates (PNE: 85.7 %; control: 88.4 %), and without differences at PD21 in mean litter size (PNE: 5.7 ± 0.7; control: 5.4 ± 0.5; t_11_ = 0.28, *p* = 0.78; *t* test), mean body weight (PNE: 7.19 ± 0.12 g; control: 7.15 ± 0.15 g; *n* = 34–38/group; *p* = 0.47; Mann–Whitney test), and mean crown-rump length (PNE: 6.12 ± 0.06 cm; control: 6.16 ± 0.08 cm; *n* = 34-38/group; *p* = 0.41; Mann–Whitney test).

### Prenatal nicotine exposure does not cause obesity or type 2 diabetes in adult first-generation offspring

**Fig. 2:**
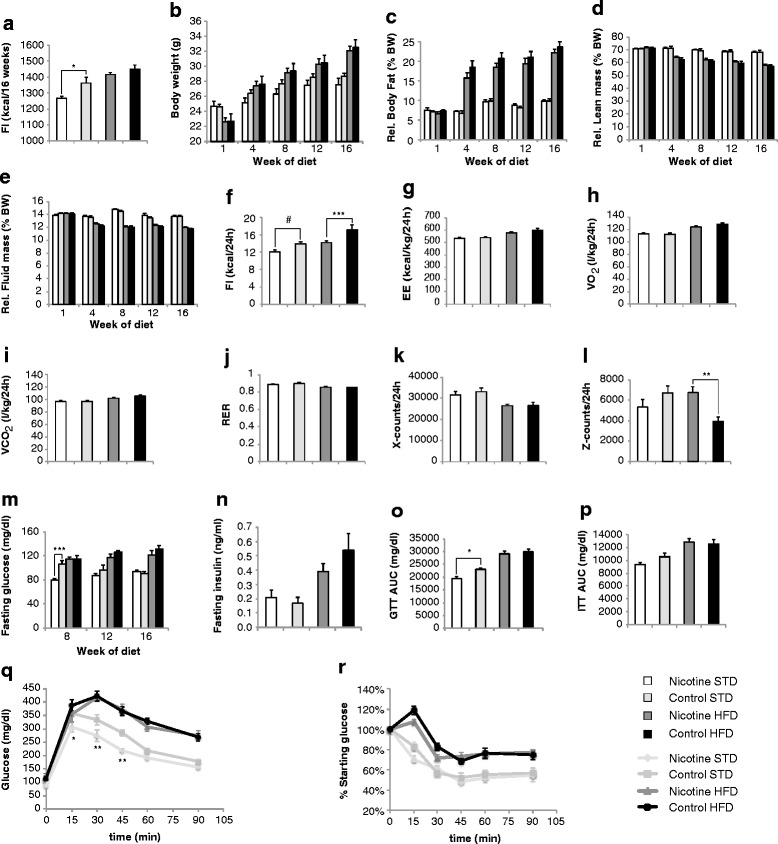
First-generation adult PNE offspring do not develop adiposity or type 2 diabetes. **a** Cumulative food intake of PNE offspring and control offspring (in kcal) was determined after 16 weeks of diet. **b** Monthly body weight measurements concomitant with body composition measurements. **c**–**e** Relative body fat (**c**), relative lean mass (**d**), and relative fluid mass (**e**) in % of body weight (BW) determined monthly by nuclear magnetic resonance. **f**–**l** metabolic cage measurements of 24-h food intake (FI) (**f**), 24-h energy expenditure (EE) (**g**), 24-h oxygen consumption rates (VO_2_) (**h**), 24-h carbon dioxide production rates (VCO_2_) (**i**), respiratory exchange ratio (RER) (**j**), and 24-h locomotor activity (**k**, **l**) in week 10 of the diets. EE, VO_2_, and VCO_2_ were normalized to the lean mass determined by nuclear magnetic resonance prior to the start of the measurements. Locomotor activity was measured as horizontal (**k**) and vertical beam breaks counts (**l**). **m** Fasting glucose. **n** Fasting insulin determined in week 16 of the diets. **o**–**p** Area under the curve (AUC) determination of Glucose tolerance tests (GTT) conducted in week 6 of the diet (**o**) and Insulin tolerance tests (ITT) conducted in week 10 of the diets (**p**). **q**–**r**, Glucose concentration measurements during the GTT (**q**) and ITT (**r**). Mice were injected i.p. glucose 1 g/kg (**q**) or Insulin 0.5 IU/kg (**r**). Data were analyzed by two-way ANOVA (**a**–**p**) or two-way RM ANOVA (**q**, **r**) followed by Bonferroni post-tests (*, *p* < 0.05; **, *p* < 0.01; ***, *p* < 0.001) or by *t* test (**f**) (^#^, *p* < 0.05). The number of male offspring in each group was *n* = 5–9 originating from at least three different dams. All data are expressed as mean ± SEM. HFD intake effectively rendered mice obese, diabetic and hypoactive (**a**–**i**, **k**–**p**: *p* < 0.005, HFD vs. STD, two-way ANOVA)

Male offspring were subjected to a 16-week STD or HFD. PNE moderately decreased cumulative food intake of STD mice (PNE: 1268 ± 9.5 kcal, *n* = 7; control: 1363 ± 37.6 kcal, *n* = 8) and HFD mice (PNE: 1416 ± 13.48 kcal, *n* = 8; control: 1450 ± 27.6 kcal, *n* = 8) (Fig. 2a). Body weight measurements revealed no differences between PNE and control offspring fed the same diet (Fig. 2b). Moreover, PNE had no impact on body composition as determined by NMR measurements of relative fat, lean, and fluid mass (Fig. 2c–e). Plasma lipid levels were profiled in week 16 of the diets and were the same in nicotine-exposed and non-exposed offspring (Table 1).

**Table 1:** Plasma lipids

Lipid (mg/dl)	Nicotine STD	Control STD	Nicotine HFD	Control HFD
Triglycerides	48.1 ± 5.4	44.6 ± 4.1	61.9 ± 5.4	67.0 ± 3.0*
Cholesterol	67.5 ± 3.1	64.7 ± 1.4	156.5 ± 12.3**	166.8 ± 4.7**
HDL-Cholesterol	50.6 ± 3.6	48.9 ± 1.3	124.3 ± 3.0**	125.9 ± 1.6**
VLDL-Cholesterol	9.6 ± 1.2	8.9 ± 0.8	12.4 ± 1.0	13.4 ± 0.6*
LDL-Cholesterol	5.2 ± 0.9	7.2 ± 1.1	27.3 ± 4.4**	27.5 ± 3.8**

Plasma lipids were determined in week 16 of the diets following 14 h overnight fasting using VITROS® technology. Data were analyzed by two-way ANOVA followed by Bonferrroni post-test (*, *p* < 0.01; **, *p* < 0.001 versus STD feeding). Each group consisted of *n* = 6–9 male offspring from at least three different dams. Data are expressed as mean ± SEM. *HDL* High density lipoprotein, *LDL* Low density lipoprotein, *VLDL* Very low density lipoprotein

To more accurately characterize PNE-induced changes in energy balance, we subjected individual male offspring in week 10 of the STD and HFD to simultaneous determinations of food intake, locomotor activity, and energy expenditure by indirect calorimetry in metabolic cages. PNE moderately reduced 24-h caloric intake of STD mice (PNE: 12.11 ± 0.38 kcal/24 h, *n* = 7; control: 13.94 ± 0.52 kcal/24 h, *n* = 7; t_12_ = 2.85 and *p* = 0.015 by *t* test) and HFD mice (PNE: 14.21 ± 0.34 kcal/24 h, *n* = 8; control: 17.16 ± 1.16 kcal/24 h, *n* = 7; F_1,25_ = 12.9 and *p* = 0.0014 by two-way ANOVA; *p* < 0.01 by Bonferroni post-test) (Fig. 2f). PNE had no impact on 24-h energy expenditure, 24-h oxygen consumption, 24-h carbon dioxide production, or the respiratory exchange rate of offspring on the STD or HFD (Fig. 2g–j). However, we noted a moderate enhancement in 24-h vertical activity of PNE offspring relative to control offspring under HFD conditions, possibly reflecting increased rearing (Fig. 2k–l). HFD feeding reduced vertical and horizontal activities in control animals relative to STD feeding (Fig. 2k–l) as we have reported earlier [6, 49].

PNE offspring displayed a transient decrease in fasting glycemia in week 8 of the STD (PNE: 80.3 ± 1.6 mg/dl, *n* = 7; control: 106.3 ± 5.3 mg/dl, *n* = 9; *p* < 0.001 by Bonferroni post-test) (Fig. 2m). Subsequent fasting glucose measurements in weeks 12 and 16 of the STD and in weeks 8, 12, and 16 of the HFD revealed no differences in fasting glycemia (Fig. 2m). A GTT conducted in week 8 of the diets revealed increased glucose tolerance in PNE offspring relative to control offspring under STD conditions (area under the curve (AUC): PNE, 19,348 ± 751 mg/dl, *n* = 7; controls, 23,069 ± 593 mg/dl, *n* = 8; *p* < 0.05 by Bonferroni post-test) but not under HFD conditions (Fig. 2o, q). An ITT conducted in week 10 of the diets showed no difference in insulin sensitivity between PNE and control groups (Fig. 2p, r). Fasting plasma insulin concentrations determined in week 16 of the diets were similar in PNE and control offspring (Fig. 2n). We conclude that PNE did not render offspring more susceptible to obesity or diabetes development but instead moderately decreased food intake and fasting glycemia.

### Prenatal nicotine exposure enhances leptin-melanocortin signaling in adult first-generation offspring

We measured the anorectic and body weight lowering actions of leptin and the MC3/4-R agonist MTII in prenatally nicotine-exposed and non-exposed adult first-generation offspring. Mice were fasted overnight, injected i.p. leptin, MTII, or vehicle (PBS), and immediately offered food, followed by determination of body weight and cumulative food consumption at defined time points [38].

**Fig. 3:**
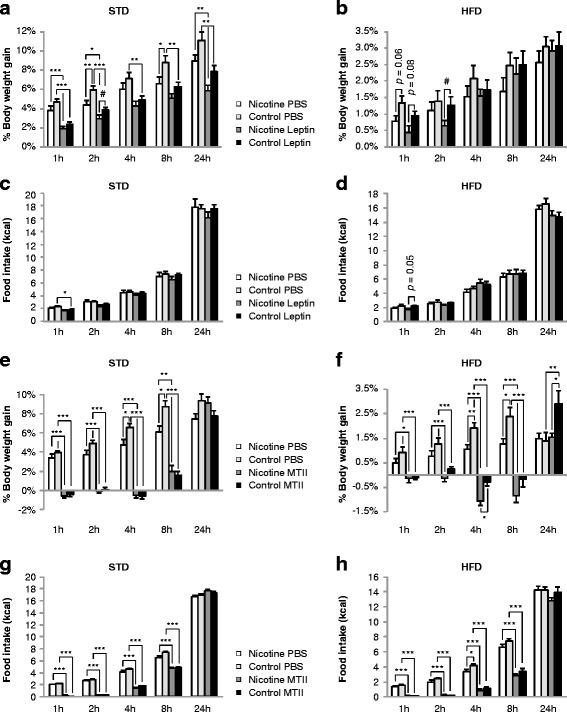
PNE decreased body weight gain and moderately increased sensitivity to leptin-induced and melanotan II (MTII)-induced body weight loss of offspring in refeeding experiments. Male offspring were fasted for 12 h, injected i.p. leptin (5 mg/kg), MTII (5 mg/kg), or vehicle (PBS), and immediately offered food. Body weight gain (% of pre-injection body weight) and food intake were determined at defined time points post injection. **a**–**d** Leptin experiments: body weight change of STD mice (**a**) and HFD mice (**b**), cumulative food intake of STD mice (**c**) and HFD mice (**d**). **e**–**h** MTII experiments: body weight change of STD mice (**e**) and HFD mice (**f**), cumulative food intake of STD mice (**g**) and HFD mice (**h**). Data were analyzed by two-way ANOVA followed by Bonferroni post-tests (*, *p* < 0.05; **, *p* < 0.01; ***, *p* < 0.001) or unpaired two-tailed *t* test: (^#^, *p* < 0.05 and numerical *p* values in **b** and **d**). The number of male offspring in each group was *n* = 7–9 originating from at least three different dams. All data are expressed as mean ± SEM

Leptin administration examined the response of first order signaling neurons controlling food intake and body weight, including hypothalamic POMC neurons. Vehicle-injected and leptin-injected PNE offspring tended to gain less body weight than vehicle-injected and leptin-injected control offspring both under STD conditions (t = 1 h: F_1,27_ = 4.71, *p* = 0.04; t = 2 h: F_1,27_ = 14.5, *p* = 0.0007; t = 8 h: F_1,27_ = 9.6, *p* = 0.005; t = 24 h: F_1,27_ = 9.9, *p* = 0.004; two-way ANOVA) (Fig. 3a) and HFD conditions (t = 1 h: F_1,28_ = 7.9, *p* = 0.009; t = 2 h: F_1,28_ = 3.3, *p* = 0.08; two-way ANOVA) (Fig. 3b). PNE offspring also tended to be more sensitive to leptin-mediated inhibition of body weight gain under STD conditions (t = 2 h: t_14_ = 1.89, *p* = 0.048; *t* test) (Fig. 3a) and HFD conditions (t = 1 h: t_14_ = 1.89, *p* = 0.079; t = 2 h: t_14_ = 2.21, *p* = 0.045; *t* test) (Fig. 3b). Moreover, vehicle-injected PNE offspring gained less body weight than vehicle-injected control offspring both on the STD (t = 2 h: *p* < 0.05; t = 8 h: *p* < 0.01; Bonferroni post-test) (Fig. 3a) and HFD (t = 1 h: t_14_ = 2.08, *p* = 0.057; *t* test) (Fig. 3b). PNE did not impact food intake of vehicle-treated and leptin-treated offspring on the STD (Fig. 3c) but moderately lowered food consumption of vehicle-treated and leptin-treated offspring on the HFD (t = 1 h: F_1,28_ = 4.6, *p* = 0.04; two-way ANOVA). Furthermore, PNE modestly increased sensitivity of HFD offspring to feeding inhibition by leptin 1 h after refeeding (t_14_ = 2.11, *p* = 0.054; *t* test) (Fig. 3d).

MTII administration examined the response of second order signaling neurons controlling food intake and body weight including those innervated by hypothalamic POMC neurons. PNE tended to decrease body weight gain of MTII-injected and vehicle-injected offspring on the STD (t = 4 h: F_1,29_ = 2.9, *p* = 0.099; t = 8 h: F_1,29_ = 2.89, *p* = 0.099; two-way ANOVA) (Fig. 3e) and HFD (t = 2 h: F_1,28_ = 5.3, *p* = 0.03; t = 4 h: F_1,28_ = 18.96, *p* < 0.0001; t = 8 h: F_1,28_ = 9.3, *p* = 0.005; two-way ANOVA) (Fig. 3f). PNE and control offspring on the STD were equally sensitive to MTII-induced body weight loss (Fig. 3e). Under HFD conditions, PNE offspring were more sensitive to MTII-induced body weight loss than control offspring (t = 4 h: *p* < 0.05; t = 24 h: *p* < 0.05; Bonferroni post-test) (Fig. 3f). As we observed during the leptin sensitivity experiments, vehicle-injected PNE offspring gained less body weight than vehicle-injected control offspring under STD conditions (t = 4 h: *p* < 0.05; t = 8 h: *p* < 0.05; Bonferroni post-test) (Fig. 3e) and HFD conditions (t = 4 h: *p* < 0.05; t = 8 h: *p* < 0.01; Bonferroni post-test) (Fig. 3f). Finally, PNE tended to moderately decrease food consumption of MTII-injected and vehicle-injected offspring on the STD (t = 4 h: F_1,29_ = 5.1, *p* = 0.03; t = 8 h: F_1,29_ = 3.8, *p* = 0.06; two-way ANOVA) (Fig. 3g) and HFD (t = 4 h: F_1,28_ = 6.2, *p* = 0.02; t = 8 h: F_1,28_ = 3.4, *p* = 0.08; two-way ANOVA) (Fig. 3h).

In summary, the refeeding experiments showed that PNE decreased body weight gain of offspring and rendered them moderately more sensitive to the acute body weight lowering effects of leptin and MTII.

**Fig. 4:**
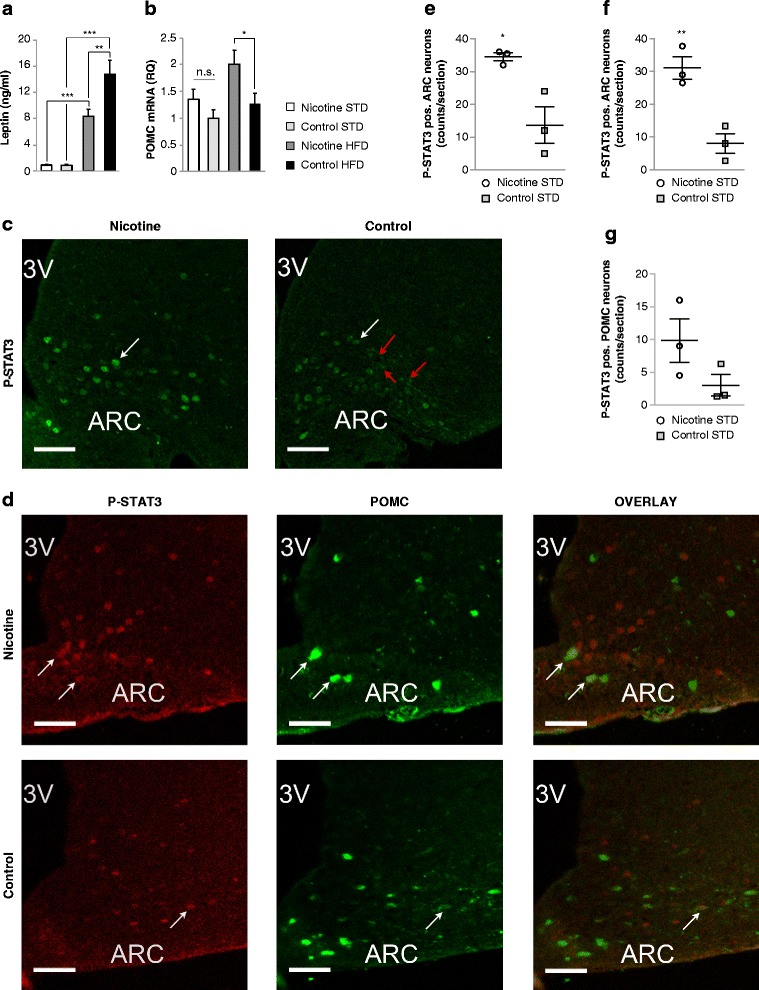
PNE enhanced hypothalamic leptin signaling in offspring. **a** Plasma leptin determined in week 16 of the diets. **b** Hypothalamic POMC mRNA levels were determined in week 16 of the diets by reverse transcription quantitative PCR, normalized to β-actin mRNA and expressed relative to the STD-fed control group. The number of male offspring in each group in (**a**) and (**b**) was *n* = 7–9 originating from at least three different dams. **c**, **d** Leptin-induced nuclear STAT3 phosphorylation (P-STAT3) of hypothalamic arcuate nucleus neurons (**c**, **d**) and hypothalamic POMC neurons (**d**) determined in STD-fed mice by immunofluorescence using antibodies against STAT3-Tyr 705 phosphorylation and POMC polypeptide. P-STAT3 was detected using either a rabbit monoclonal (**c**) or mouse monoclonal antibody (**d**, *left panel*). Male offspring were fasted for 14 h, injected i.p. leptin (5 mg/kg), and sacrificed 30 min post injection. White arrows highlight several P-STAT3 reactive nuclei in the hypothalamic arcuate nucleus (ARC) (**c**, **d**, *left panel*), POMC positive neurons (**d**, *middle panel*), and POMC positive neurons with P-STAT3 reactive nuclei (**d**, *right panel*). *Red arrows* highlight neurons with cytoplasmic P-STAT3 immunoreactivity (**c**, *right panel*). *3 V* refers to the third ventricle (**c**, **d**). The size bars represent 50 μM (**c**, **d**). **e**, **f** Quantification of leptin-induced nuclear STAT3 phosphorylation in hypothalamic arcuate neurons after staining with rabbit monoclonal P-STAT3 antibody (**e**) or mouse monoclonal P-STAT3 antibody (**f**) (*n* = 3 male offspring per group). **g** Quantification of P-STAT3 immunoreactive hypothalamic POMC neurons (*n* = 3 male offspring per group). Data were analyzed by two-way ANOVA followed by Bonferroni post-tests (*, *p* < 0.05; **, *p* < 0.01; ***, *p* < 0.001) (**a**, **b**) or by two-tailed *t* test (**e**, **f**). All data are expressed as mean ± SEM

Most human obesity and animal models of diet-induced obesity are associated with elevated plasma leptin levels [50–52], and the development of resistance to leptin’s body weight lowering actions [5]. Therefore, we measured plasma leptin concentrations as an indicator for hypothalamic leptin resistance. Plasma leptin concentrations were the same in PNE and control offspring on the STD (PNE: 0.9 ± 0.09 ng/ml, *n* = 7; control: 0.98 ± 0.18 ng/ml, *n* = 9) but they were decreased in PNE offspring under HFD conditions (PNE, 9.3 ± 1.07 ng/ml, *n* = 9; control, 14.85 ± 2.21 ng/ml, *n* = 7; *p* < 0.01 by Bonferroni post-test) (Fig. 4a). These results further suggest that PNE rendered offspring moderately more sensitive to leptin.

Leptin resistance is at least in part caused by impaired leptin signaling in the hypothalamus, including the POMC neurons [5, 52, 53]. To further examine whether PNE enhanced hypothalamic leptin signaling, we determined STAT3 phosphorylation (P-STAT3) in hypothalamic arcuate neurons 30 min after leptin i.p. injection. P-STAT3 immunoreactivity was assessed using a rabbit monoclonal (Fig. 4c) or a mouse monoclonal antibody recognizing STAT3 Tyr705 phosphorylation (Fig. 4d). P-STAT3 immunoreactivity was detected mainly in the hypothalamic arcuate nucleus (ARC) and to a minor extent in the ventromedial hypothalamic nucleus (VMH). Vehicle-injected offspring showed no P-STAT3 immunoreactivity in ARC and VMH consistent with previous observations [54, 55]. Leptin-injected PNE offspring showed a higher number of P-STAT3 positive arcuate neurons (mouse monoclonal P-STAT3 antibody: 31.1 ± 3.4/section versus 8.02 ± 3.01/section, *n* = 3, t_4_ = 5.1, *p* = 0.007, *t* test; rabbit monoclonal P-STAT3 antibody: 34.5 ± 1.3/section versus 13.7 ± 5.6/section, *n* = 3, t_4_ = 3.66, *p* = 0.02, *t* test) and tended to have a higher number of P-STAT3 positive POMC neurons (9.8 ± 3.4/section versus 3.02 ± 1.6/section, *n* = 3, t_4_ = 1.8, *p* = 0.14, *t* test) than leptin-injected control offspring (Fig. 4e, f, g). In PNE offspring, P-STAT3 reactivity was almost exclusively detected in the cell nuclei (Fig. 4c, d). In control offspring, P-STAT3 reactive nuclei were less intensely stained (Fig. 4c, d) and many cells with cytoplasmic P-STAT3 staining were detected in contrast to PNE offspring (Fig. 4c). These findings suggest that PNE offspring respond faster to leptin-induced cytoplasmic-nuclear translocation of P-STAT3 and are consistent with enhanced hypothalamic leptin-signaling in PNE offspring.

Leptin stimulates P-STAT3 mediated transcription of POMC. To further determine the long-term effects of PNE on leptin signaling, we determined the levels of hypothalamic POMC mRNA at the end of the 16-week diet. PNE moderately increased POMC mRNA of adult male offspring under HFD but not under STD conditions (F_1,28_ = 6.7, *p* = 0.015 by two-way ANOVA; *p* < 0.05 by Bonferroni post-test) (Fig. 4b) consistent with enhanced leptin-melanocortin signaling.

### Characterization of the coding and long non-coding transcriptome of hypothalamic POMC neurons

Hypothalamic POMC neurons were isolated by laser microdissection from six prenatally nicotine-exposed and five non-exposed, STD-fed adult male offspring expressing the enhanced green fluorescent protein in POMC neurons [40]. A range of 400–600 POMC neurons were isolated from each mouse brain. Strand-specific RNA-seq libraries were generated from these POMC neurons. A selective priming method during first strand cDNA synthesis minimized reverse transcription of ribosomal RNA (rRNA) sequences. Libraries were paired-end sequenced on Illumina’s HiSeq2000 sequencer. Read-pairs were aligned to the mouse reference mm10 genome using STAR [41]. Only read-pairs aligning to a unique site of the mouse genome with less than two mismatches were used for analysis. Aligned read-pairs were run through HTSeq against the GENCODE reference gtf file vM4 for determination of read-pair counts by genes under consideration of DNA strand specificity [42]. Expression of a gene was normalized by dividing the number of read-pairs mapped to its exons by the number of read-pairs mapped to the reference genome. The resulting value was expressed in counts per million (CPM). Individual expression levels of alternative transcript variants were not quantified. For all analyses, only genes with average expression values across all samples > 1 CPM were considered. Three expression calculators in edgeR [43], DESeq [44], and baySeq [45] determined differential gene expression. The intersection of differentially expressed features across these three calculators yielded the final list of differentially expressed genes.

**Fig. 5:**
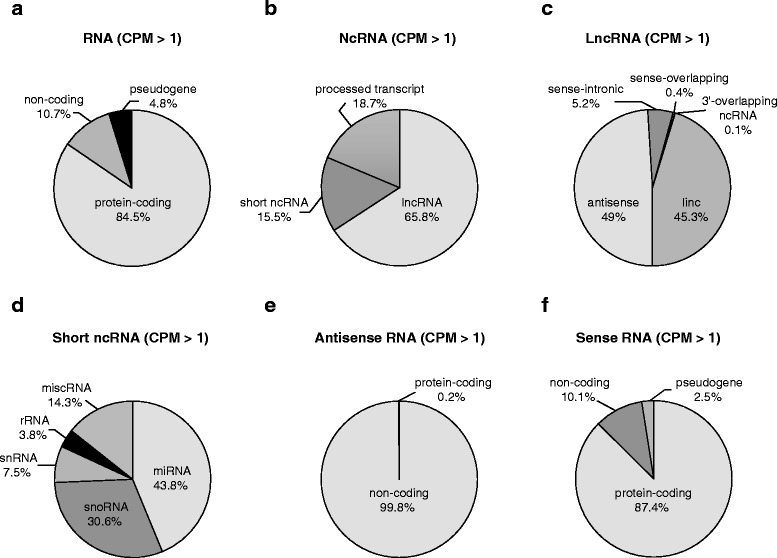
Composition of the transcriptome of hypothalamic POMC neurons by total RNA (**a**), ncRNA (**b**), lncRNA (**c**), short ncRNA (**d**), antisense RNA (**e**), and sense RNA (complementary to antisense RNA) (**f**). Only RNAs expressed at levels > 1 CPM were considered for analyses

Each library yielded on average 42.9 million read-pairs passing Illumina’s quality control filters (PNE: 46.3 million; control: 38.8 million). On average, 30 million or 70.5 % read-pairs per library were aligned to unique sites of the reference genome (PNE: 32.7 million or 71.3 %; Control: 26.8 million or 69.4 %) (Table 2). Read-pairs mapped to 16,014 genes with average expression levels > 1 CPM (Additional file 1: Table S1). Of these, 13,539 (84.5 %) were protein-coding genes, 1708 (10.7 %) were ncRNA genes, and 767 (4.8 %) were pseudogenes (Fig. 5a). NcRNA genes comprised 1124 (65.8 %) lncRNA genes, 265 (15.5 %) short ncRNA genes, and 319 (18.7 %) processed transcripts (Fig. 5b). Mapped lncRNA genes comprised 509 (45.3 %) lincRNA genes, 551 (49 %) antisense RNA genes, 59 (5.2 %) sense-intronic RNA genes, four (0.4 %) sense-overlapping RNA genes, and one (0.1 %) 3′-overlapping ncRNA gene (Fig. 5c). Mapped short ncRNA genes were composed of 116 (43.8 %) microRNA (miRNA) genes, 81 (30.6 %) small nucleolar RNA (snoRNAs) genes, 20 (7.5 %) small nuclear RNA (snRNA) genes, ten (3.8 %) rRNA genes, and 38 (14.3 %) miscellaneous RNA (miscRNA) genes (Fig. 5d). The fraction of expressed short ncRNA genes is likely underestimated because the RNA isolation procedure excluded RNAs smaller than 100 nucleotides. Antisense RNA genes expressed at levels > 1 CPM were almost exclusively non-coding (551 or 99.8 %) and overlapped 529 (87.4 %) protein-coding genes, 61 (10.1 %) ncRNA genes, and 15 (2.5 %) pseudogenes (Fig. 5f).

**Table 2:** RNA-seq alignment statistics

Sample	Input read-pairs	Uniquely mapped read-pairs	Uniquely mapped read-pairs (%)
N1	39,371,421	28,694,853	72.9
N2	33,778,273	23,411,127	69.3
N3	37,980,952	26,573,624	70.0
N4	43,271,579	31,630,187	73.1
N5	98,021,209	67,023,419	68.4
N6	25,381,274	18,901,075	74.5
C1	43,903,675	28,076,176	63.9
C2	31,996,428	22,462,184	70.2
C3	39,669,137	26,617,656	67.1
C4	31,587,579	23,443,335	74.2
C5	46,728,455	33,418,612	71.5
Average	42,880,907	30,022,932	70.5
Average nicotine (*n* = 6)	46,300,785	32,705,714	71.3
Average control (*n* = 5)	38,777,055	26,803,593	69.4

Six PNE offspring (denoted as N1–N6) and five control offspring (denoted as C1–C5) were paired-end sequenced on Illuminas HiSeq2000 sequencer. Read-pairs passing Illumina’s internal quality filters (Input read-pairs) were aligned to the mouse mm10 reference genome using STAR [41]. Read-pairs mapped to a unique site of the mouse mm10 reference genome with less than two mismatches (Uniquely mapped read-pairs) were used for further analyses

**Fig. 6:**
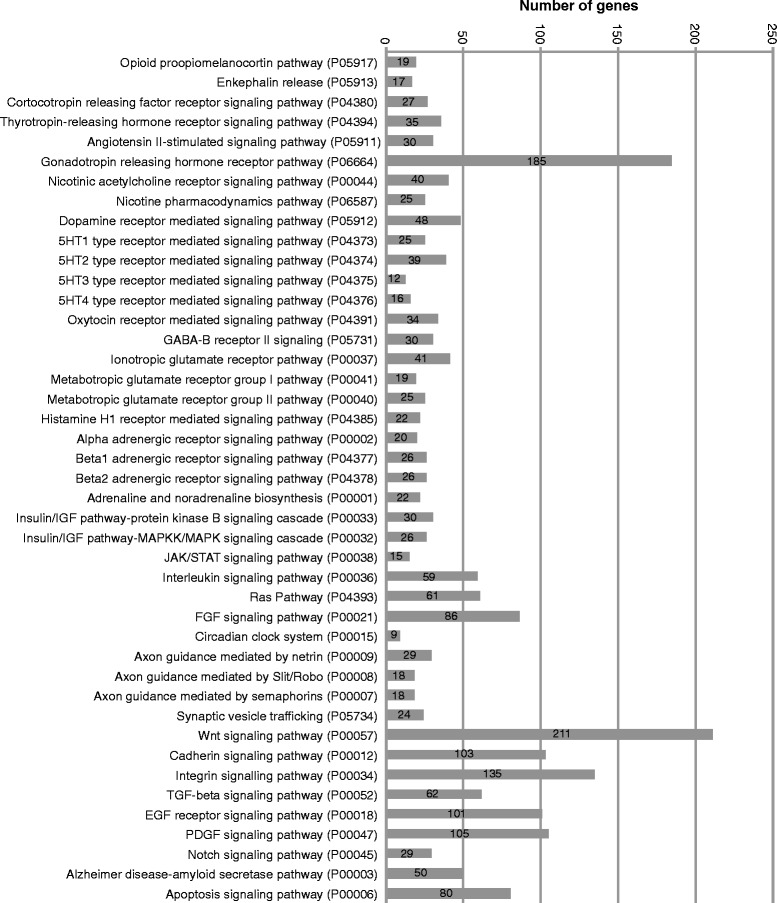
Assignment of expressed genes to PANTHER defined pathways. The PANTHER pathway accession numbers and the number of mapped genes are indicated. Only genes expressed at levels > 1 CPM were considered

A total of 13,534 protein-coding genes with average expression levels > 1 CPM were subjected to gene ontology (GO) classification using the PANTHER gene analysis tool [46]. PANTHER classified 12,731 protein-coding genes by molecular function, biological process, protein class, cellular component, and pathway. Most genes had binding (3817; 33.1 %), catalytic (3763; 32.7 %), and transcription factor activity (1097; 9.5 %) (Table 3), were involved in a metabolic (5775; 50.1 %) and cellular process (3721; 32.3 %) (Table 4), encoded nucleic acid binding proteins (1703; 14.8 %) and transcription factors (1110; 9.6 %) (Table 5), and were associated with organelles (679; 5.9 %) and macromolecular complexes (437; 3.8 %) (Table 6). GO classification by pathway detected the existence of numerous signaling and neurodevelopment pathways in hypothalamic POMC neurons (Fig. 6; Table 7; Additional file 2: Table S2). As expected, we found expression of 19 and 17 genes participating in the opioid proopiomelanocortin and enkephalin release pathways, respectively (Additional file 2: Table S2). Moreover, RNA-seq revealed expression of 40 genes participating in the nicotinic acetylcholine receptor signaling pathway including genes encoding the neuronal acetylcholine receptor subunit α-4 (Chrna4), consistent with a previous report [10], the neuronal acetylcholine receptor subunit α -1 (Chrna1), α-7 (Chrna7), β-1 (Chrnb1), β-2 (Chrnb2), and acetylcholinesterase (Ache) (Additional file 2: Table S2).

**Table 3:** Gene Ontology classification of protein-coding genes by molecular function

Category name (GO accession number)	Number of gene hits	Percent of gene hit against total number of genes	Percent of gene hit against total number of function hits
Antioxidant activity (GO:0016209)	18	0.20 %	0.10 %
Binding (GO:0005488)	3817	33.10 %	31.70 %
Catalytic activity (GO:0003824)	3763	32.70 %	31.20 %
Enzyme regulator activity (GO:0030234)	717	6.20 %	5.90 %
Nucleic acid binding transcription factor activity (GO:0001071)	1097	9.50 %	9.10 %
Protein binding transcription factor activity (GO:0000988)	147	1.30 %	1.20 %
Receptor activity (GO:0004872)	849	7.40 %	7.00 %
Structural molecule activity (GO:0005198)	786	6.80 %	6.50 %
Translation regulator activity (GO:0045182)	116	1.00 %	1.00 %
Transporter activity (GO:0005215)	745	6.50 %	6.20 %

PANTHER classified 12,731 protein-coding genes with average expression values > 1 CPM by molecular function. A total of 13,395 molecular functions were hit

**Table 4:** Gene Ontology classification of protein-coding genes by biological process

Category name (GO accession number)	Number of gene hits	Percent of gene hit against total number of genes	Percent of gene hit against total number of process hits
Apoptotic process (GO:0006915)	421	3.70 %	2.10 %
Biological adhesion (GO:0022610)	556	4.80 %	2.80 %
Biological regulation (GO:0065007)	2073	18.00 %	10.40 %
Cellular component organization or biogenesis (GO:0071840)	781	6.80 %	3.90 %
Cellular process (GO:0009987)	3721	32.30 %	18.70 %
Developmental process (GO:0032502)	1648	14.30 %	8.30 %
Growth (GO:0040007)	3	0.00 %	0.00 %
Immune system process (GO:0002376)	872	7.60 %	4.40 %
Localization (GO:0051179)	1779	15.40 %	9.00 %
Locomotion (GO:0040011)	7	0.10 %	0.00 %
Metabolic process (GO:0008152)	5775	50.10 %	29.10 %
Multicellular organismal process (GO:0032501)	1065	9.20 %	5.40 %
Reproduction (GO:0000003)	306	2.70 %	1.50 %
Response to stimulus (GO:0050896)	841	7.30 %	4.20 %

PANTHER classified 12,731 protein-coding genes with average expression values > 1 CPM by biological process. A total of 21,970 biological processes were hit

**Table 5:** Gene Ontology classification of protein-coding genes by protein class

Category name (PANTHER protein class accession number)	Number of gene hits	Percent of gene hit against total number of genes	Percent of gene hit against total number of protein class hits
Calcium-binding protein (PC00060)	242	2.10 %	1.90 %
Cell adhesion molecule (PC00069)	310	2.70 %	2.50 %
Cell junction protein (PC00070)	104	0.90 %	0.80 %
Chaperone (PC00072)	168	1.50 %	1.30 %
Cytoskeletal protein (PC00085)	541	4.70 %	4.30 %
Defense/immunity protein (PC00090)	238	2.10 %	1.90 %
Enzyme modulator (PC00095)	981	8.50 %	7.90 %
Extracellular matrix protein (PC00102)	289	2.50 %	2.30 %
Hydrolase (PC00121)	1046	9.10 %	8.40 %
Isomerase (PC00135)	116	1.00 %	0.90 %
Kinase (PC00137)	400	3.50 %	3.20 %
Ligase (PC00142)	350	3.00 %	2.80 %
Lyase (PC00144)	131	1.10 %	1.00 %
Membrane traffic protein (PC00150)	316	2.70 %	2.50 %
Nucleic acid binding (PC00171)	1703	14.80 %	13.60 %
Oxidoreductase (PC00176)	404	3.50 %	3.20 %
Phosphatase (PC00181)	227	2.00 %	1.80 %
Protease (PC00190)	337	2.90 %	2.70 %
Receptor (PC00197)	872	7.60 %	7.00 %
Signaling molecule (PC00207)	506	4.40 %	4.10 %
Storage protein (PC00210)	9	0.10 %	0.10 %
Structural protein (PC00211)	75	0.70 %	0.60 %
Surfactant (PC00212)	28	0.20 %	0.20 %
Transcription factor (PC00218)	1110	9.60 %	8.90 %
Transfer/carrier protein (PC00219)	263	2.30 %	2.10 %
Transferase (PC00220)	943	8.20 %	7.60 %
Transmembrane receptor regulatory/adaptor protein (PC00226)	58	0.50 %	0.50 %
Transporter (PC00227)	715	6.20 %	5.70 %
Viral protein (PC00237)	3	0.00 %	0.00 %
Storage protein (PC00210)	9	0.10 %	0.10 %
Structural protein (PC00211)	75	0.70 %	0.60 %
Urfactant (PC00212)	28	0.20 %	0.20 %
Transcription factor (PC00218)	1110	9.60 %	8.90 %
Transfer/carrier protein (PC00219)	263	2.30 %	2.10 %
Transferase (PC00220)	943	8.20 %	7.60 %
Transmembrane receptor regulatory/adaptor protein (PC00226)	58	0.50 %	0.50 %
Transporter (PC00227)	715	6.20 %	5.70 %
Viral protein (PC00237)	3	0.00 %	0.00 %
Storage protein (PC00210)	9	0.10 %	0.10 %
Structural protein (PC00211)	75	0.70 %	0.60 %
Surfactant (PC00212)	28	0.20 %	0.20 %
Storage protein (PC00210)	9	0.10 %	0.10 %
Structural protein (PC00211)	75	0.70 %	0.60 %
Surfactant (PC00212)	28	0.20 %	0.20 %
Transcription factor (PC00218)	1110	9.60 %	8.90 %
Transfer/carrier protein (PC00219)	263	2.30 %	2.10 %
Transferase (PC00220)	943	8.20 %	7.60 %
Transmembrane receptor regulatory/adaptor protein (PC00226)	58	0.50 %	0.50 %
Transporter (PC00227)	715	6.20 %	5.70 %
Viral protein (PC00237)	3	0.00 %	0.00 %

PANTHER classified 12,731 protein-coding genes with average expression values > 1 CPM by protein class. A total of 13,887 protein classes were hit

**Table 6:** Gene Ontology classification of protein-coding genes by cellular component

Category name (GO accession number)	Number of gene hits	Percent of gene hit against total number of genes	Percent of gene hit against total number of cellular component hits
Cell junction (GO:0030054)	47	0.40 %	1.50 %
Cell part (GO:0044464)	1063	9.20 %	34.90 %
Extracellular matrix (GO:0031012)	229	2.00 %	7.50 %
Extracellular region (GO:0005576)	314	2.70 %	10.30 %
Macromolecular complex (GO:0032991)	437	3.80 %	14.40 %
Membrane (GO:0016020)	272	2.40 %	8.90 %
Organelle (GO:0043226)	679	5.90 %	22.30 %
Synapse (GO:0045202)	4	0.00 %	0.10 %

PANTHER classified 12,731 protein-coding genes with average expression values > 1 CPM by cellular component. Protein-coding genes hit a total of 3366 cellular components

**Table 7:** Gene Ontology classification of protein-coding genes by pathway

Category name (PANTHER pathway accession number)	Number of gene hits	Percent of gene hit against total number of genes	Percent of gene hit against total number of pathway hits
2-arachidonoylglycerol biosynthesis (P05726)	4	0.00 %	0.10 %
5-Hydroxytryptamine biosynthesis (P04371)	2	0.00 %	0.00 %
5-Hydroxytryptamine degradation (P04372)	3	0.00 %	0.10 %
5HT1 type receptor mediated signaling pathway (P04373)	25	0.20 %	0.60 %
5HT2 type receptor mediated signaling pathway (P04374)	39	0.30 %	1.00 %
5HT3 type receptor mediated signaling pathway (P04375)	12	0.10 %	0.30 %
5HT4 type receptor mediated signaling pathway (P04376)	16	0.10 %	0.40 %
Acetate utilization (P02722)	3	0.00 %	0.10 %
Activin-beta signaling pathway (P06210)	3	0.00 %	0.10 %
Adenine and hypoxanthine salvage pathway (P02723)	6	0.10 %	0.10 %
Adrenaline and noradrenaline biosynthesis (P00001)	22	0.20 %	0.50 %
Alanine biosynthesis (P02724)	2	0.00 %	0.00 %
ALP23B_signaling_pathway (P06209)	3	0.00 %	0.10 %
Alpha adrenergic receptor signaling pathway (P00002)	20	0.20 %	0.50 %
Alzheimer disease-amyloid secretase pathway (P00003)	50	0.40 %	1.20 %
Alzheimer disease-presenilin pathway (P00004)	74	0.60 %	1.80 %
Aminobutyrate degradation (P02726)	2	0.00 %	0.00 %
Androgen/estrogene/progesterone biosynthesis (P02727)	3	0.00 %	0.10 %
Angiogenesis (P00005)	125	1.10 %	3.10 %
Angiotensin II-stimulated signaling through G proteins and beta-arrestin (P05911)	30	0.30 %	0.70 %
Apoptosis signaling pathway (P00006)	80	0.70 %	2.00 %
Arginine biosynthesis (P02728)	4	0.00 %	0.10 %
Ascorbate degradation (P02729)	2	0.00 %	0.00 %
Asparagine and aspartate biosynthesis (P02730)	4	0.00 %	0.10 %
ATP synthesis (P02721)	6	0.10 %	0.10 %
Axon guidance mediated by netrin (P00009)	29	0.30 %	0.70 %
Axon guidance mediated by semaphorins (P00007)	18	0.20 %	0.40 %
Axon guidance mediated by Slit/Robo (P00008)	18	0.20 %	0.40 %
B cell activation (P00010)	49	0.40 %	1.20 %
Beta1 adrenergic receptor signaling pathway (P04377)	26	0.20 %	0.60 %
Beta2 adrenergic receptor signaling pathway (P04378)	26	0.20 %	0.60 %
Beta3 adrenergic receptor signaling pathway (P04379)	11	0.10 %	0.30 %
Blood coagulation (P00011)	22	0.20 %	0.50 %
BMP_signaling_pathway-drosophila (P06211)	2	0.00 %	0.00 %
Cadherin signaling pathway (P00012)	103	0.90 %	2.50 %
Carnitine and CoA metabolism (P02732)	1	0.00 %	0.00 %
Carnitine metabolism (P02733)	1	0.00 %	0.00 %
Cell cycle (P00013)	17	0.10 %	0.40 %
Cholesterol biosynthesis (P00014)	9	0.10 %	0.20 %
Circadian clock system (P00015)	9	0.10 %	0.20 %
Coenzyme A biosynthesis (P02736)	6	0.10 %	0.10 %
Cortocotropin releasing factor receptor signaling pathway (P04380)	27	0.20 %	0.70 %
Cysteine biosynthesis (P02737)	1	0.00 %	0.00 %
Cytoskeletal regulation by Rho GTPase (P00016)	57	0.50 %	1.40 %
De novo purine biosynthesis (P02738)	19	0.20 %	0.50 %
De novo pyrimidine deoxyribonucleotide biosynthesis (P02739)	6	0.10 %	0.10 %
De novo pyrmidine ribonucleotides biosythesis (P02740)	8	0.10 %	0.20 %
DNA replication (P00017)	19	0.20 %	0.50 %
Dopamine receptor mediated signaling pathway (P05912)	48	0.40 %	1.20 %
DPP_signaling_pathway (P06213)	2	0.00 %	0.00 %
DPP-SCW_signaling_pathway (P06212)	2	0.00 %	0.00 %
EGF receptor signaling pathway (P00018)	101	0.90 %	2.50 %
Endogenous_cannabinoid_signaling (P05730)	18	0.20 %	0.40 %
Endothelin signaling pathway (P00019)	65	0.60 %	1.60 %
Enkephalin release (P05913)	17	0.10 %	0.40 %
FAS signaling pathway (P00020)	24	0.20 %	0.60 %
FGF signaling pathway (P00021)	86	0.70 %	2.10 %
Flavin biosynthesis (P02741)	2	0.00 %	0.00 %
Folate biosynthesis (P02742)	2	0.00 %	0.00 %
Formyltetrahydroformate biosynthesis (P02743)	4	0.00 %	0.10 %
Fructose galactose metabolism (P02744)	9	0.10 %	0.20 %
GABA-B_receptor_II_signaling (P05731)	30	0.30 %	0.70 %
Gamma-aminobutyric acid synthesis (P04384)	5	0.00 %	0.10 %
GBB_signaling_pathway (P06214)	2	0.00 %	0.00 %
General transcription by RNA polymerase I (P00022)	13	0.10 %	0.30 %
General transcription regulation (P00023)	30	0.30 %	0.70 %
Glutamine glutamate conversion (P02745)	2	0.00 %	0.00 %
Glycolysis (P00024)	18	0.20 %	0.40 %
Gonadotropin releasing hormone receptor pathway (P06664)	185	1.60 %	4.60 %
Hedgehog signaling pathway (P00025)	19	0.20 %	0.50 %
Heme biosynthesis (P02746)	8	0.10 %	0.20 %
Heterotrimeric G-protein signaling pathway-Gi alpha and Gs alpha mediated pathway (P00026)	103	0.90 %	2.50 %
Heterotrimeric G-protein signaling pathway-Gq alpha and Go alpha mediated pathway (P00027)	86	0.70 %	2.10 %
Heterotrimeric G-protein signaling pathway-rod outer segment phototransduction (P00028)	17	0.10 %	0.40 %
Histamine H1 receptor mediated signaling pathway (P04385)	22	0.20 %	0.50 %
Histamine H2 receptor mediated signaling pathway (P04386)	9	0.10 %	0.20 %
Huntington disease (P00029)	112	1.00 %	2.80 %
Hypoxia response via HIF activation (P00030)	21	0.20 %	0.50 %
Inflammation mediated by chemokine and cytokine signaling pathway (P00031)	135	1.20 %	3.30 %
Insulin/IGF pathway-mitogen activated protein kinase kinase/MAP kinase cascade (P00032)	26	0.20 %	0.60 %
Insulin/IGF pathway-protein kinase B signaling cascade (P00033)	30	0.30 %	0.70 %
Integrin signalling pathway (P00034)	135	1.20 %	3.30 %
Interferon-gamma signaling pathway (P00035)	24	0.20 %	0.60 %
Interleukin signaling pathway (P00036)	59	0.50 %	1.50 %
Ionotropic glutamate receptor pathway (P00037)	41	0.40 %	1.00 %
Isoleucine biosynthesis (P02748)	3	0.00 %	0.10 %
JAK/STAT signaling pathway (P00038)	15	0.10 %	0.40 %
Leucine biosynthesis (P02749)	2	0.00 %	0.00 %
Lipoate_biosynthesis (P02750)	2	0.00 %	0.00 %
Mannose metabolism (P02752)	5	0.00 %	0.10 %
Metabotropic glutamate receptor group I pathway (P00041)	19	0.20 %	0.50 %
Metabotropic glutamate receptor group II pathway (P00040)	25	0.20 %	0.60 %
Metabotropic glutamate receptor group III pathway (P00039)	45	0.40 %	1.10 %
Methionine biosynthesis (P02753)	2	0.00 %	0.00 %
Methylcitrate cycle (P02754)	2	0.00 %	0.00 %
Methylmalonyl pathway (P02755)	3	0.00 %	0.10 %
mRNA splicing (P00058)	6	0.10 %	0.10 %
Muscarinic acetylcholine receptor 1 and 3 signaling pathway (P00042)	38	0.30 %	0.90 %
Muscarinic acetylcholine receptor 2 and 4 signaling pathway (P00043)	29	0.30 %	0.70 %
P53 pathway feedback loops 1 (P04392)	5	0.00 %	0.10 %
P53 pathway feedback loops 2 (P04398)	41	0.40 %	1.00 %
Parkinson disease (P00049)	74	0.60 %	1.80 %
PDGF signaling pathway (P00047)	105	0.90 %	2.60 %
Pentose phosphate pathway (P02762)	7	0.10 %	0.20 %
Phenylethylamine degradation (P02766)	1	0.00 %	0.00 %
PI3 kinase pathway (P00048)	40	0.30 %	1.00 %
Plasminogen activating cascade (P00050)	3	0.00 %	0.10 %
PLP biosynthesis (P02759)	1	0.00 %	0.00 %
Proline biosynthesis (P02768)	4	0.00 %	0.10 %
Purine metabolism (P02769)	7	0.10 %	0.20 %
Pyridoxal phosphate salvage pathway (P02770)	1	0.00 %	0.00 %
Pyrimidine Metabolism (P02771)	8	0.10 %	0.20 %
Pyruvate metabolism (P02772)	6	0.10 %	0.10 %
Ras Pathway (P04393)	61	0.50 %	1.50 %
S adenosyl methionine biosynthesis (P02773)	2	0.00 %	0.00 %
Salvage pyrimidine deoxyribonucleotides (P02774)	1	0.00 %	0.00 %
Salvage pyrimidine ribonucleotides (P02775)	6	0.10 %	0.10 %
SCW_signaling_pathway (P06216)	2	0.00 %	0.00 %
Serine glycine biosynthesis (P02776)	5	0.00 %	0.10 %
Succinate to proprionate conversion (P02777)	2	0.00 %	0.00 %
Sulfate assimilation (P02778)	2	0.00 %	0.00 %
Synaptic_vesicle_trafficking (P05734)	24	0.20 %	0.60 %
T cell activation (P00053)	58	0.50 %	1.40 %
TCA cycle (P00051)	9	0.10 %	0.20 %
TGF-beta signaling pathway (P00052)	62	0.50 %	1.50 %
Thiamine metabolism (P02780)	3	0.00 %	0.10 %
Threonine biosynthesis (P02781)	2	0.00 %	0.00 %
Thyrotropin-releasing hormone receptor signaling pathway (P04394)	35	0.30 %	0.90 %
Toll receptor signaling pathway (P00054)	39	0.30 %	1.00 %
Toll_pathway_drosophila (P06217)	1	0.00 %	0.00 %
Transcription regulation by bZIP transcription factor (P00055)	41	0.40 %	1.00 %
Triacylglycerol metabolism (P02782)	1	0.00 %	0.00 %
Tryptophan biosynthesis (P02783)	1	0.00 %	0.00 %
Tyrosine biosynthesis (P02784)	1	0.00 %	0.00 %
Ubiquitin proteasome pathway (P00060)	50	0.40 %	1.20 %
Valine biosynthesis (P02785)	3	0.00 %	0.10 %
Vasopressin synthesis (P04395)	11	0.10 %	0.30 %
VEGF signaling pathway (P00056)	47	0.40 %	1.20 %
Vitamin B6 metabolism (P02787)	3	0.00 %	0.10 %
Vitamin D metabolism and pathway (P04396)	7	0.10 %	0.20 %
Wnt signaling pathway (P00057)	211	1.80 %	5.20 %
Xanthine and guanine salvage pathway (P02788)	4	0.00 %	0.10 %

PANTHER classified 12,731 protein-coding genes with average expression values > 1 CPM by pathway. A total of 4445 pathways were hit

We also detected expression of various G-protein coupled receptor-signaling pathways that were previously reported to regulate feeding and body weight, to be expressed in POMC neurons and/or to regulate POMC neuronal activity. These included the dopamin receptor [56], oxytocin receptor [57], metabotropic glutamate receptor I-III [58, 59], ionotropic glutamate receptor, histamine H1/H2 receptor [60], serotonin 1–4 (5HT1-4) receptor [61], GABA-B receptor II [62, 63], α-adrenoceptor [64], β1-3 adrenoceptor, and muscarinic acetylcholine receptor 1–4 pathways [65] (Fig. 6; Table 7; Additional file 2: Table S2).

POMC neurons expressed a synaptic vesicle trafficking pathway (Fig. 6; Table 7; Additional file 2: Table S2), the vesicular glutamate transporter 2 (vGlut2 or Slc17a6) and vesicular GABA transporter (vGat or Slc32a1), mediating uptake of glutamate and GABA/glycine into synaptic vesicles, respectively (Additional file 1: Table S1).

Moreover, we found expression of the insulin/insulin like growth factor (Igf) /protein kinase B (Akt), insulin/Igf/mitogen activated protein kinase kinase (Mapkk)/mitogen activated protein kinase (Mapk), and Jak/Stat signaling pathways in hypothalamic POMC neurons (Fig. 6; Table 7; Additional file 2: Table S2). RNA-seq also revealed expression of three key genes of the leptin receptor signaling pathway, the leptin receptor (*Lepr*), *Jak2* and *Stat3* genes, and regulators of the leptin signaling pathway including suppressor of cytokine signaling 3 (*Socs3*) and protein tyrosine phosphatase 1 b (*Ptpn1*) (Additional file 1: Table S1).

POMC neurons also expressed various neuroendocrine-signaling pathways. These included the thyrotropin-releasing hormone (TRH) receptor, cortocotropin releasing factor (CRF) receptor, and gonadotropin releasing hormone (GnRH) receptor signaling pathways (Fig. 6; Table 7; Additional file 2: Table S2). Moreover, we found expression of the fibroblast growth factor (FGF) signaling pathway and several fibroblast growth factors including Fgf1, Fgf9, Fgf12, Fgf13, and Fgf14 (Fig. 6; Table 7; Additional file 2: Table S2). POMC neurons also expressed several genes encoding core components of the circadian clock (Fig. 6; Table 7; Additional file 2: Table S2).

POMC neurons further expressed several neurodevelopment pathways. These included the netrin, Slit/Robo, and semaphorin axon guidance pathways, as well as the wnt, cadherin, integrin, transforming growth factor (TGF)-β, platelet derived growth factor (PDGF), and epidermal growth factor (EGF) pathways (Fig. 6; Table 7; Additional file 2: Table S2). Finally, RNA-seq detected expression of the Notch signaling and Alzheimer amyloid secretase pathways (Fig. 6; Table 7; Additional file 2: Table S2).

We ranked 1119 lncRNA genes (509 lincRNA, 551 antisense RNA, and 59 sense-intronic RNA genes) expressed at levels > 1 CPM by expression (Additional file 3: Table S3). Of these, *Yam1*, *Malat1*, *Meg3*, *Gm26870*, *Gm15564*, *6330403K07Rik*, *Kcnq1ot1*, *Miat*, *A330023F24Rik*, and *Gm14703* were the most abundantly expressed lncRNAs (Additional file 3: Table S3). LncRNAs can regulate coding genes *in cis* through epigenetic, transcriptional, and post-transcriptional mechanism. To gain explorative insight into potential gene regulatory functions of lncRNAs expressed in POMC neurons, we assessed linear co-expression of lncRNAs (antisense RNA, lincRNA, sense-intronic RNA) with their nearest (adjacent or overlapping) protein-coding gene applying an expression threshold of 1 CPM for both, coding and non-coding gene expression. A Pearson’s correlation coefficient *r* > 0.602 or < −0.602 (*n* = 11, *p* < 0.05 by two-tailed *t* test) was regarded as significant. A total of 1053 lncRNA genes (452 lincRNA, 548 antisense RNA, and 53 sense-intronic RNA genes) and 991 protein-coding genes forming 1096 lncRNA/coding gene pairs were examined. We detected 82 co-expressed lncRNA/coding gene pairs comprising 41 antisense RNA/coding gene pairs, 34 lincRNA/coding gene pairs, and seven sense-intronic RNA/coding gene pairs (Additional file 4: Table S4). 59 (or 72 %) lncRNA/protein-coding gene pairs were positively co-expressed whereas 23 (or 28 %) were negatively (discordantly) co-expressed. In over 75 % of the co-expressed lncRNA/coding gene pairs, expression level of the lncRNA was less than 30 % that of the coding gene. In the majority, 65 or 79 %, of lncRNA/coding gene pairs, the lncRNA overlapped the coding gene. Furthermore, in 29 or 35 % of lncRNA/coding gene pairs, the lncRNA overlapped the coding gene promoter spanning the 10 kb sequence upstream of its first exon (Additional file 4: Table S4).

Since lncRNAs can be transcribed from enhancers as so-called enhancer RNAs (eRNAs) to stimulate gene transcription [66, 67], we determined whether lncRNAs that were co-expressed with their nearest protein-coding genes were transcribed from conserved mouse enhancers annotated in the VISTA enhancer browser database [68]. Only one lncRNA, *Gm16263*, an antisense RNA to the co-expressed protein-coding gene *Fgf10*, was transcribed from an annotated mouse enhancer. This enhancer spanned a region 1089 nucleotides upstream and 313 nucleotides downstream of the transcription start site of *Fgf10* (mm10 build, chr13:118713610–118715012), thus overlapping the *Fgf10* gene promoter and a small fragment of exon1 of *Fgf10*.

Co-expressed protein-coding genes had functions in nervous system development (*Irx3*, *Msi2*, *Lhx1*, *Sox4*, *Plcb1*), neurite outgrowth (*Farp1*, *Lhx1*, *Ppp1r9a*, *Rapgef4*, *Srgap2*), synaptogenesis (*Cep112*, *Farp1*, *Fgf10*, *Myo6*), neurotransmission (*Hrh3*, *Gabra5*, *Plcb1*, *Rapgef4*, *Myo6*, *Kcna3*, *Kcna6*, *Kcnmb4*), intracellular Ca^2+^ signaling (*Pitpnm2*, *Slc8a3*, *Plcb1*), insulin signaling (*Irs4*, *Plcb1*, *Aspscr1*, *Baiap2l1*), mitochondrial biogenesis, ubiquitin-dependent protein degradation, transcription regulation, RNA processing, cell cycle, cell division, cell growth, cell differentiation, cell adhesion, organization of the cytoskeleton, and apoptosis (Additional file 4: Table S4). Nineteen lncRNAs (*A330102I10Rik*, *B930095G15Rik*, *Cep112os2*, *C030037D09Rik*, *Gm2727*, *Gm9962*, *Gm16006*, *Gm16263*, *Gm17180*, *Gm26673*, *Gm26814*, *Gm27008*, *Irx3os*, *Kcnmb4os1*, *Lhx1os*, *Rapgef4os3*, *RP24-302 F18.2*, *9630028H03Rik*, *2210017G18Rik*) were co-expressed with the above-mentioned coding genes regulating nervous system development and function (Additional file 4: Table S4).

### Prenatal nicotine upregulates *Gm15851* in adult offspring hypothalamic POMC neurons

**Fig. 7:**
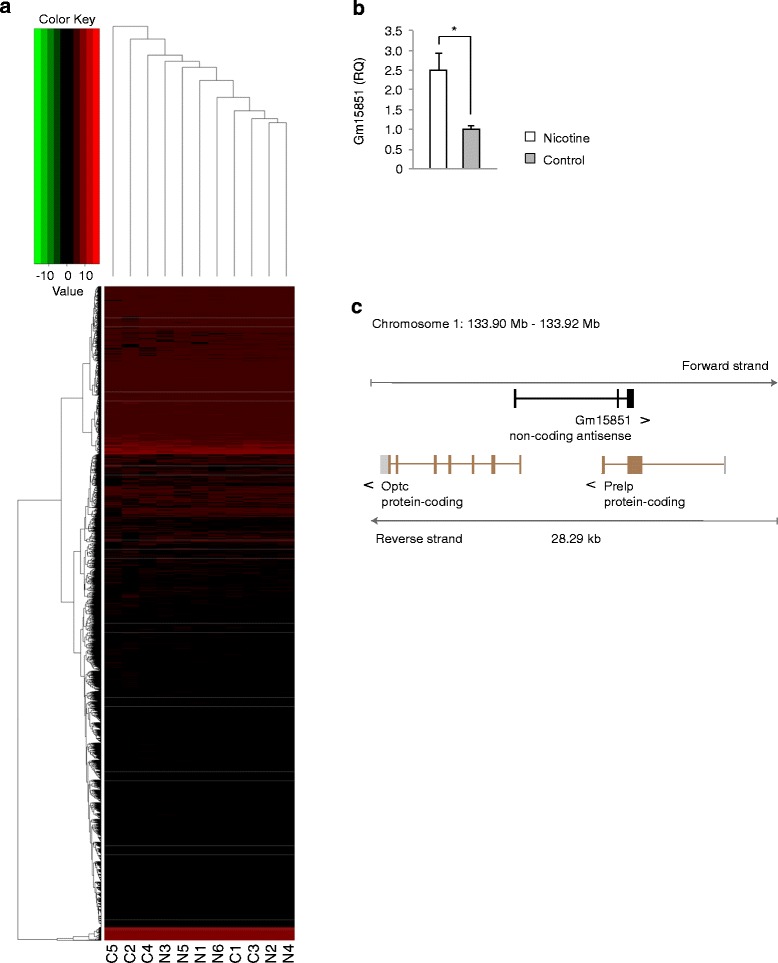
PNE upregulated an antisense RNA, *Gm15851*, in hypothalamic POMC neurons of adult offspring. **a** *Two-way clustering* of RNA-seq expression data of POMC neurons reveal overlapping gene expression signatures in PNE offspring (N1–N6) and control offspring (C1–C5). The *color code* represents the gene expression value in log_2_CPM. Only genes with average expression levels > 1 CPM across all offspring were considered. **b** Strand-specific qRT-PCR confirms overexpression of *Gm15851* in POMC neurons of PNE offspring (*, *p* < 0.05, *n* = 5–6, two-tailed *t* test with Welch correction). **c** Schematic of genomic localization of *Gm15851* within a 28.29 kb segment spanning the chromosomal coordinates 133.9 Mb and 133.92 Mb of chromosome 1. *Gm15851* is a spliced antisense transcript to *Prelp* and *Optc* on the forward strand of mouse chromosome 1. *Boxes* denote exons. *Arrowheads* denote gene orientation

The intersection of the results of three differential expression calculators, edgeR, baySeq, and DESeq, revealed only one consistent expression difference between nicotine-exposed and non-exposed offspring, an 80-fold upregulation of *Gm15851* in PNE offspring (FDR-adjusted *p* value: 0.006 by edgeR, 0.002 by baySeq and DESeq; average expression level: 4.5 CPM). Two-way clustering revealed overlapping gene expression signatures of prenatally nicotine-exposed and non-exposed hypothalamic POMC neurons (Fig. 7a). Quantitative reverse transcription polymerase chain reaction (qRT-PCR) confirmed upregulation of *Gm15851* in POMC neurons of nicotine-exposed offspring (t_5_ = 3.17, *p* = 0.025 by *t* test with Welch’s correction) (Fig. 7b). *Gm15851* is a lncRNA of 662 nucleotides encoded by three exons on the forward strand of chromosome 1 (Fig. 7c). Supporting evidence for the existence of this transcript comes from one expressed sequence tag in the adult mouse male epididymis cDNA RIKEN full-length enriched library (RIKEN clone 9230112 L10) [21]. *Gm15851* lacks a significant open reading frame. It is an antisense RNA overlapping two protein-coding genes, opticin (*Optc*) and proline arginine-rich end leucine-rich repeat (*Prelp*), on the reverse strand. The third exon of *Gm15851* overlaps the second coding exon of *Prelp* whereas the first exon of *Gm15851* overlaps the first intron of *Optc* (Fig. 7c). Although *Optc* and *Prelp* are potential targets of *cis*-regulation by *Gm15851*, both genes were not co-expressed with *Gm15851* in POMC neurons.

## Discussion

Contrary to expectations based on previous studies of gestational and neonatal nicotine exposure and energy balance, we report here that gestational nicotine exposure does not render offspring more susceptible to diet-induced obesity or type 2 diabetes. Instead, we found that gestational nicotine exposure moderately decreased food intake, body weight gain, and glycemia, and mildly increased sensitivity to leptin and melanocortin receptor stimulation in first-generation adult offspring. Consistent with a moderate enhancement in leptin sensitivity, PNE offspring were more sensitive to leptin-induced STAT3 phosphorylation, and—under HFD conditions—had increased POMC mRNA and decreased plasma leptin concentrations. Consistent with previous reports [35, 69], PNE offspring displayed increased voluntary locomotor activity under HFD conditions, which could reflect augmented exploratory behavior [69] or might result from enhanced central leptin-melanocortinergic signaling via POMC neurons as others and we have reported [6, 70]. Interestingly, although food intake was moderately decreased and voluntary locomotion moderately increased under HFD feeding conditions, overall energy balance was unaffected in PNE offspring since neither body weights nor body fat content nor energy expenditure were measurably changed. It is possible that altered central regulation of body temperature in PNE offspring accounts for this outcome. Impaired central activation of brown fat thermogenesis could lead to compensatory hyperlocomotion to increase heat expenditure and preserve body temperature. Yet another possibility is that metabolic efficiency—the degree to which ingested food is being metabolized to perform work and generate heat—could be increased in PNE offspring. These alternative mechanisms require further investigation.

Furthermore, it remains to be clarified whether PNE has sexually dimorphic effects on the regulation of energy balance in offspring. Recently, Burke et al. reported that a subpopulation of hypothalamic arcuate POMC neurons, which express 5-hydroxytryptamine 2c receptors (5-HT2CRs) and account for 40 % of all hypothalamic arcuate POMC neurons, regulates energy balance in a sexually dimorphic manner [71]. The authors re-expressed POMC selectively in 5-HT2CR containing POMC neurons of POMC-deficient obese and insulin-resistant mice. Re-expression of POMC in this neuronal subpopulation was sufficient to normalize food intake, physical activity, brown fat heat expenditure, body weight, adiposity, and insulin sensitivity in males. Meanwhile, re-expression of POMC in this neuronal subpopulation in females corrected only food intake and insulin sensitivity but did neither increase physical activity, energy expenditure nor block the development of obesity [71].

Previous human epidemiological and meta-analysis studies [11–14] reported a positive correlation between PNE and the risk for obesity and type 2 diabetes in children and young adults. This correlation was further supported by rat studies of gestational and lactational nicotine exposure [15–18]. The onset, length, and route of nicotine administration likely determine the cumulative dose and developmental stage at which hypothalamic POMC neuronal progenitor cells are exposed to nicotine. Maternal plasma cotinine levels in the present and in previous rodent studies reporting metabolic disturbances in the offspring were of similar magnitude [15–17], suggesting that dams were exposed to comparable doses of nicotine. However, previous studies reporting diabetes in the offspring administered nicotine subcutaneously to dams [15–17, 72]. Nicotine that is subcutaneously administered to dams may reach the fetus at a higher concentration than maternally ingested nicotine because the former does not undergo first-pass hepatic conversion to cotinine contrary to the latter. In addition to differences in the route of administration, gestational nicotine exposure was continued throughout lactation in two studies reporting diabetes and obesity in the offspring [15, 17]. Bruin et al. [72] showed that disturbances in glucose homeostasis in the offspring required nicotine exposure to occur both during gestation and lactation. This would be in line with the timing of development of murine hypothalamic arcuate neurons, which differentiate between embryonic days 10 and 16 [73] and form projections in the first three postnatal weeks to other hypothalamic regions involved in feeding regulation [74, 75]. Our observations suggest dose-dependent and time-dependent effects of nicotine on developing hypothalamic POMC neurons. Exposure to nicotine at a low dose during gestation might stimulate proliferation, differentiation, neurite outgrowth, and synaptogenesis of developing hypothalamic POMC neurons, thereby sensitizing leptin-melanocortinergic signaling in adult offspring, whereas exposure to nicotine at a high dose during gestation and lactation might impair those processes, leading to the reported obesity and diabetes phenotypes in adult offspring.

RNA-seq of hypothalamic POMC neurons isolated from adult PNE and control offspring followed by differential expression analyses using three expression calculators detected only one consistent change, a nicotine-induced upregulation of *Gm15851*, an antisense RNA to the *Prelp* and *Optc* genes. Although *Gm15851* was not co-expressed with *Prelp* or *Optc* in terminally differentiated POMC neurons, we cannot exclude that *cis*-regulatory interactions between *Gm15851* and *Prelp* or *Optc* exist in POMC neuronal progenitor cells. LncRNAs may serve as scaffolds for gene-specific recruitment of transcription factors, transcriptional complexes, and/or chromatin-modifying complexes during specific developmental time windows. These additional regulators may not be expressed in terminally differentiated POMC neurons thus preventing regulatory interactions of *Gm15851* with *Optc* and/or *Prelp* to be seen. It is possible that, in addition to regulation of transcription or chromatin state, *Gm15851* might regulate post-transcriptional processes such as splicing, editing, translation, or localization of the *Prelp* or *Optc* RNA. Interestingly, *Prelp* and *Optc* both encode extracellular matrix proteins of the family of leucine-rich repeat (LRR) domain-containing proteins. LRR domain containing proteins have been documented to organize axon guidance, target selection, and synapse formation [76]. Hence, it might be possible that *Gm15851* could modulate the establishment of synaptic connections of POMC neuronal progenitor cells by regulating expression of *Prelp* and/or *Optc*.

Whole transcriptome sequencing of hypothalamic POMC neurons confirmed the expected expression of the Opioid proopiomelanocortin release pathways, known G-protein coupled receptor pathways regulating feeding and body weight, and the insulin receptor and leptin receptor signaling pathways. Furthermore, we confirmed expression of several nicotinic acetylcholine receptor subunits and acetylcholine metabolizing enzymes in POMC neurons [10]. We also detected expression of specific transport proteins (vGlut2 and vGat) required for the uptake of glutamate and GABA/glycine into synaptic vesicles, supporting the notion that POMC neurons release glutamate and GABA [77, 78].

Several neuroendocrine signaling pathways including those mediated by the GnRH, TRH, and CRF receptors were also expressed in POMC neurons. While POMC neurons regulate reproduction, energy expenditure and stress responses through activation of hypothalamic GnRH-producing, TRH-producing, and CRF-producing neurons, respectively, the reciprocal regulation of POMC neurons by these hormonal signaling pathways remains to be explored.

POMC neurons further expressed the netrin, slit, and semaphorin axon guidance pathways. Netrin, slit, and semaphorin are secreted or membrane-bound chemotropic proteins that attract or repulse growing axons and migrating neural progenitor cells by binding, respectively, to the unc, neuropilins, and plexin transmembrane receptors [79]. These proteins guide migration and neuronal outgrowth of hypothalamic oxytocin, antidiuretic hormone, and GnRH-producing neurons [80–82]. Furthermore, the slit/robo and semaphorin/plexin/neuropilin pathways are expressed during development of the hypothalamic PVN [83], an important projection site of POMC neurons. These cell migration and axon guidance pathways could therefore regulate migration of POMC neurons and formation of connections to relevant target sites.

In addition, we found expression of the wnt, integrin, cadherin, PDGF, EGF, and Notch signaling pathways in POMC neurons, all of which have been linked to the development of hypothalamic neurons. The wnt and integrin signaling pathways regulate differentiation of hypothalamic neuronal progenitor cells [84] and migration of hypothalamic GnRH producing neurons [85], respectively. Cadherin signaling regulates neuronal connectivity and wiring of hypothalamic POMC neurons [86, 87]. The Notch signaling pathway regulates differentiation of hypothalamic arcuate neural progenitor cells into POMC and AgRP neurons [88].

Furthermore, hypothalamic POMC neurons expressed an Alzheimer amyloid secretase and TGF-β signaling pathway, which included expression of several TGF-β encoding genes (TGF-β1, TGF-β2, TGF-β3). Both pathways have been proposed to be disease-relevant. Excessive production of TGF-β by POMC neurons was found to promote hypothalamic inflammation and type 2 diabetes in obesity and during aging [89]. Expression of an Alzheimer amyloid secretase pathway suggests participation of POMC neurons in the pathogenesis of diabetic comorbidities in Alzheimer’s disease [90].

We attempted to gain insight into probable biological functions of expressed lncRNAs based on predicted *cis*-regulatory interactions with protein-coding genes. This approach revealed 82 co-expressed lncRNA/coding gene pairs, 19 of which involved coding genes regulating neural development and/or function. Most co-expressed lncRNAs and coding genes overlapped each other. In around 35 % of co-expressed lncRNA/coding gene pairs, the lncRNA also overlapped the coding gene promoter. These observations suggest that some of these lncRNAs could regulate chromatin state and/or promoter activity of the coding gene. Furthermore, we found that only one of the co-expressed lncRNAs was transcribed from an annotated conserved mouse enhancer. Such eRNAs are known to mediate gene-specific transcription by facilitating enhancer-promoter interactions, recruiting transcription factors, and blocking enhancer-binding of gene repressive factors [66, 67]. Several co-expressed coding genes had annotated functions in nervous system development such as *Irx3*, *Msi2* and *Ppp1r9a. Irx3* is a homeobox transcription factor that is expressed in the prospective neural plate in a subset of neural precursor cells and possibly regulates specification of neural progenitor cells [91]. *Msi1* is a neural RNA-binding protein that is highly enriched in neural precursor cells and drives proliferation of neurons and glial cells in the CNS during embryonic development [92], postnatally and in adults [93]. *Ppp1r9a*, also referred to as neurabin, is selectively expressed in neural tissues, where it induces F-actin cross-linking activity in the synapse and lamellipodia of the growth cone to regulate neurite formation [94].

## Conclusions

Gestational nicotine exposure may not cause obesity and type 2 diabetes in first-generation offspring but instead may moderately enhance central leptin-melanocortinergic regulation of energy and glucose balance via POMC neurons. Gestational nicotine exposure upregulates *Gm15851*, a lncRNA, which might modulate POMC neuronal development and/or function. POMC neurons express 82 *cis*-regulatory lncRNA/protein-coding gene interactions, 19 of which involve coding genes regulating neural development and/or function, and several previously unidentified metabolic, neuroendocrine, and neurodevelopment pathways.

## Additional files

Additional file 1: Table S1. Gene list. Read-pairs were aligned by STAR to the mouse reference genome. STAR alignments were run through HTSeq for determination of read-pair counts by genes. Genes are denoted by their Ensembl gene ID, gene symbol, and gene type (columns A–C). EdgeR calculated average gene expression values across all offspring (column D) and fold-changes between nicotine-exposed and control offspring (FC (N/C), column E). EdgeR (column F), baySeq (column G), and DESeq (column H) determined differential gene expression. *FDR* false discovery rate-adjusted *p* value, *CPM* counts per million. (XLSX 2364 kb) Additional file 2: Table S2. Expressed protein-coding genes in PANTHER pathways. Expressed protein-coding genes denoted by Ensembl Gene ID (column A), gene name and symbol (column B), Panther family/subfamily (column C), Panther protein class (column D), and average expression in CPM (column E) were assigned to PANTHER defined pathways, which are referred to by name and accession number. Only genes expressed at levels > 1 CPM were considered for analysis. *CPM* counts per million. (XLSX 172 kb) Additional file 3: Table S3. Expression ranking of lncRNAs in POMC neurons. LncRNA genes referred to by Ensembl Gene ID (column A), gene name (column B), and gene type (column C) were ranked by average expression across all samples (column D). Only lncRNA genes expressed at levels > 1 CPM were considered for analysis. *CPM* counts per million. (XLSX 98 kb) Additional file 4: Table S4. Co-expressed lncRNAs and protein-coding genes *in cis.* Co-expression of lncRNAs (columns A and B) with their nearest (adjacent or overlapping) protein-coding gene (column D) was determined by the Pearson’s correlation coefficient *r* (column F). An *r* value > 0.602 or < −0.602 was regarded as significant (*p* < 0.05, two-tailed t test, *n* = 11). Co-expressed lncRNA/protein-coding gene pairs are ranked by the absolute *r* value. A positive *r* value indicates positive correlation and a negative *r* value indicates negative correlation (column G). The distance between lncRNA and protein-coding gene (column H) was defined as the distance between their nearest borders irrespective of strand orientation (columns C, E) and was set 0 for overlapping lncRNAs and protein-coding genes. LncRNAs were positioned upstream, overlapping, and/or downstream of the coding gene body (column I). A number of lncRNAs overlapped the coding gene promoter (column J) defined as the 10 kb upstream sequence of a gene. Only lncRNA and coding genes with average expression levels > 1 CPM (columns K and L) were considered for analyses. LncRNA expression was in most cases less than that of the coding gene (column M). Coding genes function in various biological processes (column N). (XLSX 63 kb)

## Abbreviations

AgRP: Agouti-related protein
CRF: Cortocotropin-releasing factor
EGF: Epidermal growth factor
FDR: False discovery rate
FGF: Fibroblast growth factor
GnRH: Gonadotropin-releasing hormone
GTT: Glucose tolerance test
HFD: High-fat diet
ip: Intraperitoneal
ITT: Insulin tolerance test
Kb: Kilobases
lincRNA: long intergenic non-coding RNA
lncRNA: long non-coding RNA
MC3/4-Rs: Melanocortin 3/4 receptors
MTII: Melanotan II
ncRNA: non-coding RNA
NMR: Nuclear magnetic resonance
NPY: Neuropeptide Y
PBS: Phosphate buffered saline
PD: Postnatal day
PDGF: Platelet-derived growth factor
PNE: Prenatal nicotine exposure
POMC: Proopiomelanocortin
PVN: Periventricular nucleus
RM ANOVA: Repeated measures ANOVA
RNA-seq: RNA sequencing
RRID: Research resource identifier
STAT3: Signal transducer and activator of transcription 3
STD: Standard (low-fat) diet
TGF-β: Transforming growth factor β
TRH: Thyrotropin-releasing hormone
VMH: Ventromedial hypothalamic nucleus
α-MSH: α-melanocyte-stimulating hormone
